# Fusion/fission protein family identification in Archaea

**DOI:** 10.1128/msystems.00948-23

**Published:** 2024-05-03

**Authors:** Anastasiia Padalko, Govind Nair, Filipa L. Sousa

**Affiliations:** 1 Genome Evolution and Ecology Group, Department of Functional and Evolutionary Ecology, University of Vienna, Vienna, Austria; 2 Vienna Doctoral School of Ecology and Evolution, University of Vienna, Vienna, Austria; LifeMine Therapeutics, Cambridge, Massachusetts, USA

**Keywords:** large-scale screening, comparative genomics, archaeal evolution, archaeal biology, bacterial fusions

## Abstract

The majority of newly discovered archaeal lineages remain without a cultivated representative, but scarce experimental data from the cultivated organisms show that they harbor distinct functional repertoires. To unveil the ecological as well as evolutionary impact of *Archaea* from metagenomics, new computational methods need to be developed, followed by in-depth analysis. Among them is the genome-wide protein fusion screening performed here. Natural fusions and fissions of genes not only contribute to microbial evolution but also complicate the correct identification and functional annotation of sequences. The products of these processes can be defined as fusion (or composite) proteins, the ones consisting of two or more domains originally encoded by different genes and split proteins, and the ones originating from the separation of a gene in two (fission). Fusion identifications are required for proper phylogenetic reconstructions and metabolic pathway completeness assessments, while mappings between fused and unfused proteins can fill some of the existing gaps in metabolic models. In the archaeal genome-wide screening, more than 1,900 fusion/fission protein clusters were identified, belonging to both newly sequenced and well-studied lineages. These protein families are mainly associated with different types of metabolism, genetic, and cellular processes. Moreover, 162 of the identified fusion/fission protein families are archaeal specific, having no identified fused homolog within the bacterial domain. Our approach was validated by the identification of experimentally characterized fusion/fission cases. However, around 25% of the identified fusion/fission families lack functional annotations for both composite and split states, showing the need for experimental characterization in Archaea.

**IMPORTANCE:**

Genome-wide fusion screening has never been performed in *Archaea* on a broad taxonomic scale. The overlay of multiple computational techniques allows the detection of a fine-grained set of predicted fusion/fission families, instead of rough estimations based on conserved domain annotations only. The exhaustive mapping of fused proteins to bacterial organisms allows us to capture fusion/fission families that are specific to archaeal biology, as well as to identify links between bacterial and archaeal lineages based on cooccurrence of taxonomically restricted proteins and their sequence features. Furthermore, the identification of poorly characterized lineage-specific fusion proteins opens up possibilities for future experimental and computational investigations. This approach enhances our understanding of Archaea in general and provides potential candidates for in-depth studies in the future.

## INTRODUCTION

Recent advancements in sequencing techniques have revolutionized our ability to explore the prokaryotic world, especially Archaea (1–4), by providing vast amounts of metagenomic data at a cost-effective rate. Despite the daily deposition of thousands of metagenome-assembled genomes (MAGs) in public databases (5), the isolation and cultivation of these newly discovered microbial lineages remains challenging and time-consuming (6–10). In the absence of cultivated representatives, the functional repertoire of the discovered lineages remains largely unknown, with metabolic predictions being made based only on their genomic repertoire. Archaea are of particular interest due to their less studied biology as compared to Bacteria (11), with many unknowns to be discovered.

Unlike Bacteria, Archaea were discovered much later, in the 1970s (12), and were recognized as a separate domain of life in the 1990s (13). They thrive in diverse habitats and often dominate in extreme environments (11). Their cultivation, similar to some bacteria, imposes some challenges such as long generation times and metabolite limitations in the absence of syntropic/symbiotic partners outside of their natural habitat (6, 8, 9, 14). As a consequence, the isolation and cultivation of pure cultures and co-cultures for Archaea have been successful only for a few high-rank lineages out of the 24 known phyla (15). Moreover, microbiologists have been working on bacteria longer than with archaea which can also explain why there are more bacterial isolates than archaeal ones. However, existing (meta)genome-based metabolic reconstructions (8, 16–20), growth experiments (21–24), and experimental validation of key proteins (25–28) have allowed us to estimate the notable archaeal impact on carbon, nitrogen, and sulfur cycles, three major biogeochemical cycles on early and modern Earth (11).

Computational approaches are routinely used to decipher microbial physiology and evolution within MAGs (29–34), with comparative genomics methods remaining the state-of-the-art for homology-driven functional assignments. Well-established homology detection methods entail structure/sequence similarity searches (e.g., Dali, BLAST, HMMER) (35–38), context methods (gene synteny, ontology) (39), and reciprocal best BLAST hits with Markov Clustering algorithms (40) to link sequences to known functions. However, homologous families often contain both orthologs and paralogs, whose functions can be quite diverse (41). Furthermore, evolutionary rates vary between and within protein families such that floating thresholds are required to group homologs or separate them into their respective orthologs. Nevertheless, functional predictions can still rely on orthology detection methods.

Genomic reorganizations and fusion of functionally linked genes, in particular, impose yet another challenge on homology detection (42). The Rosetta stone method (43–45) for the identification of gene fusions states that two or more genes (split genes) encoded in one genome can be found together (composite gene) in the same genome or any other. The process of gene merging corresponds to a fusion event, while the opposite process of gene splitting corresponds to a fission event. This can hinder the grouping of proteins into their natural ortholog families since a fused protein can have higher sequence similarity to one of its split counterparts, with the other marked as absent. Hence, fusions although providing undeniable functional clues, also complicate the sorting of proteins into their natural families.

Using small data sets of (mainly) closed bacterial genomes, previous studies have identified fusion/fission events in prokaryotic databases, revealing that fusion events are more common within the same functional category (42), as a result of modular genome organization common for prokaryotes (46, 47) and that the rate of fusion events is at least four times higher than fissions (42). Also, direct correlations between genome size and number of fusion events were proposed (45, 48). These studies were pivotal to establish the baseline criteria for fusion/fission events identification, their evaluation in prokaryotes and moreover, support the hypothesis of complex protein families’ evolution through fusions of smaller ones (49). More importantly, they show that fusion identification can be a tool to aid in phylogenetic and metabolic reconstructions. As genomic data expands, so do the attempts to integrate fusion predictions systematically, for instance, in the case of STRING (50), SEED (51), and the Integrated Microbial Genomes (IMG) databases (52). So far, these predictive annotations relied exclusively on hidden Markov model (HMM) assignments and on genomic colocalization criteria which can lead to false-positive (FP) identifications. To reduce false positives, in more recent studies (45, 53), a system of rigorous model coverage cutoffs, together with attempts to remove large families of promiscuous domains as fusion partners was implemented.

Herein, we integrated local alignment searches and HMM profiling, alongside genomic colocalization features, to analyze an extensive data set of archaeal genomes. Unlike previous studies that focused solely on archaeal model organisms, our screening includes uncultivated lineages to enhance the understanding of archaeal functional diversity. The identified fusion families were then compared with a large bacterial data set to explore potential evolutionary scenarios. In addition, our results were cross-referenced with existing predictions and experimentally characterized fusions to validate our findings. This comprehensive approach provides valuable insights into the evolution and functional diversity of fusion and fission events in archaeal genomes.

## RESULTS

**Fig 1:**
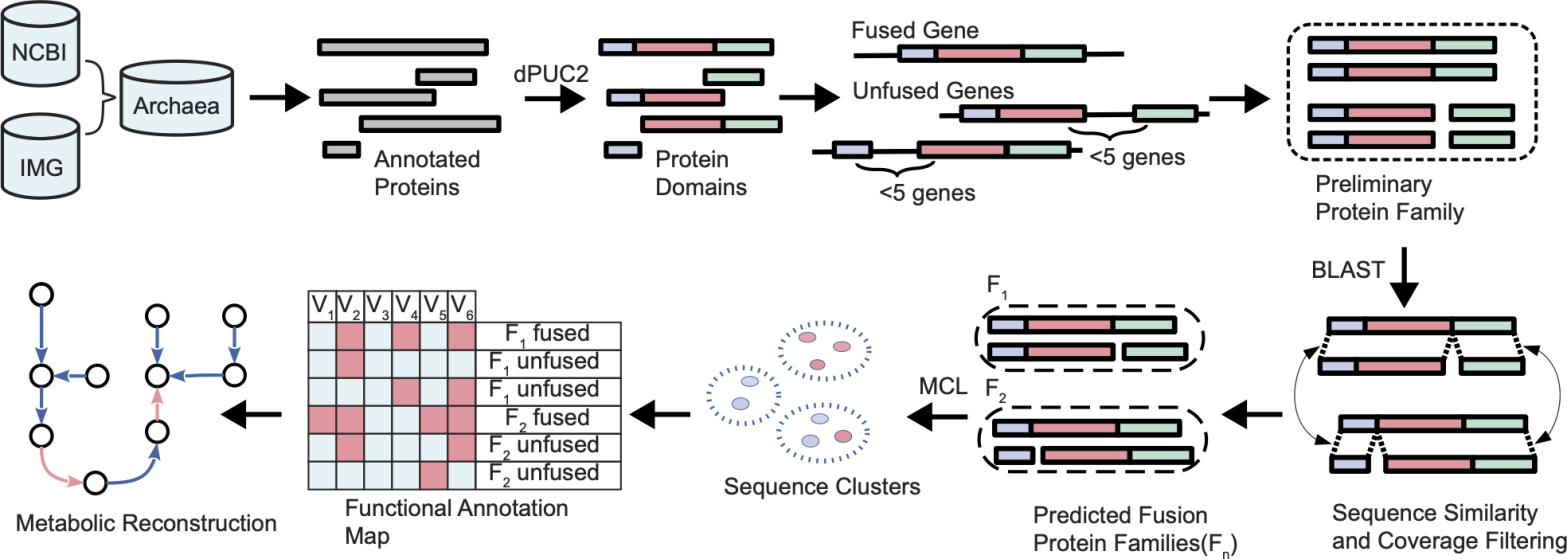
Fusion/fission protein screening pipeline.

A predictive pipeline to screen for fusion/fission proteins was developed and applied to a data set of 1,678 archaeal genomic assemblies (Table S1). The predictions were based on several criteria (see Materials and Methods). Briefly, the genomic assemblies were run against the PFAM database and the initial candidate protein fusions were defined as the ones having two or more non-overlapping PFAM domains. Based on the Rosetta stone method, the protein fusion candidate (composite protein) would only be retained if, within the genomic data set, the corresponding split protein set (matching each of the single domains of the initial composite candidate) was identified. Given that fusion/fission events tend to occur between functionally related proteins (46, 47), often encoded in proximity to each other, we implemented a genomic colocalization criterion for at least one split protein set per candidate family. Finally, at least 60% of the composite protein had to be covered by the respective split components (Fig. 1).

**Fig 2:**
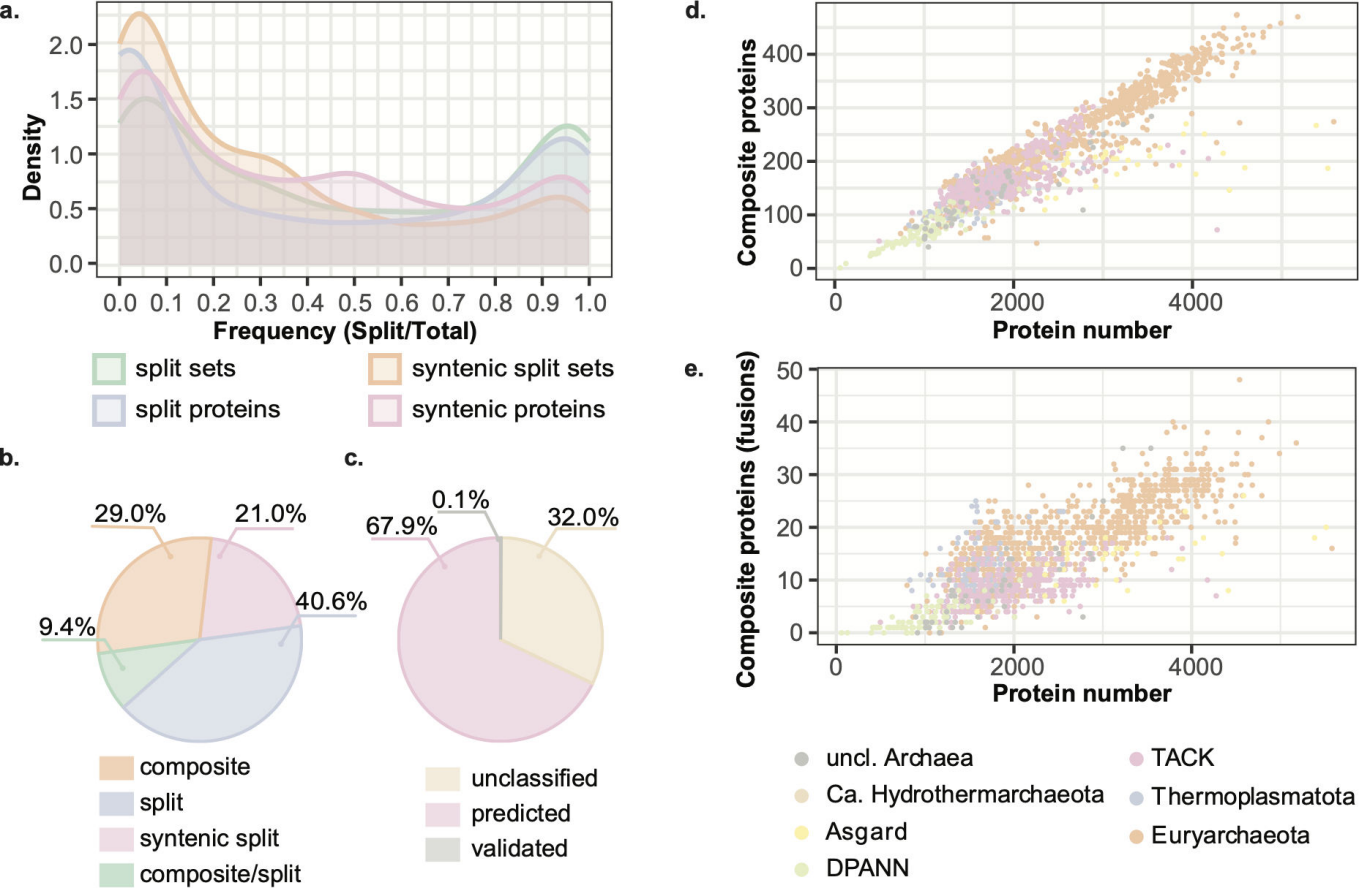
Composite (fused) and split (unfused) protein quantification. (**a**) Frequency distribution (smoothed density) between composite and split proteins, split syntenic/nonsyntenic sets, and composite proteins. (**b)**) Proportion of composite and split proteins identified. (c) Proportion of validated function (BRENDA or SwissProt), predicted function (significant KO), and unclassified. (**d**) Correlation between the number of composite proteins and the number of proteins per assembly. (**e)**) Correlation between the total number of fusion proteins and the number of proteins per assembly.

As a result, we obtained 2,183 fusion/fission protein families grouped by domain architecture. The predicted composite proteins were clustered into 1,927 clusters, with five clusters further divided based on the assigned functional annotations (e.g., 14_1). this approach led to a total of 1,932 clusters. As clusters can contain multiple domain architectures, a cluster subindex was assigned for each one of those (e.g., 1.1, Table S2). Of those, 558 only contained a single protein (singletons, Table S2) and were excluded from classification (see below). The frequency of syntenic split protein sets in regard to the total number of composite and syntenic split sets per family shows a left-skewed distribution with a weak sign for bimodality (right orange peak, Fig. 2a). Since in every split set at least two proteins are present (versus only one in the composite side), the frequency distribution considering the total number of syntenic proteins (instead of sets) is shifted to the right by 0.2. (Fig. 2a, in pink). At the same time, the frequency of total split proteins and the corresponding split sets indicates no skew for either of the two modes (Fig. 2a, in blue and green). Despite split proteins accounting for 61.6% of proteins within our data set, the total number of composite proteins almost doubles the number of the exclusively syntenic ones (Fig. 2b). The functional annotation of the proteins revealed that although experimentally characterized proteins comprise only 0.1% of the protein data set, almost 70% of protein families had KEGG orthology (KO) annotations (Fig. 2c; Table S2) enriching metabolic reconstructions. The composite proteins were used to screen the bacterial data set (Table S3) for proteins with identical domain composition and a set of ~12 million composite bacterial proteins was obtained (see Materials and Methods and supplemental table deposit at reference 54.

### Caveats

This screening strategy relies on sequence similarity, domain identifications, as well as syntenic analysis. Thus, several artifacts can mask the results and those will be discussed below. It is possible that throughout time, homologous sequences diverged beyond the level of 25% identity, defined as our cut-off. Also, some of the proteins might fail below PFAM model thresholds and thus, have no PFAM assignment. So, some cases might have not been identified by the method. However, our comparison with experimentally validated archaeal fusions and the ones reported in a fusion study in *E. coli* (45) has shown that in the majority of reported cases (Table S4), the composite and/or split components were identified. Some exceptions are for instance, the case of leader peptidase HopD where the N-terminus is not covered by PFAM models, or in the case of the bifunctional enzyme Fae/Hps (55), where no single Hps domain was found. In addition, the method does not consider homology at the family level and the hits are assigned to the most similar (higher identity) protein. An example would be the case of the fusion of the siroheme domain with the F_420_ domain as happens in the F_420_-dependent sulfite reductase, Fsr, found in methanogens (56) which, although homologous to DsrA and DsrB proteins from the Dsr-dependent dissimilatory sulfur metabolism (57), has higher local homology to the AsrC(Dsr-LP group III) and thus was attributed to this family. This closer relationship can also be observed in the phylogenies of the entire family (57). Moreover, we have imposed a synteny filtering, in which, a fusion/fission protein is only kept if, in at least one genome, the split components are within five genes from each other. Although in biology, genomic rearrangements are common, the probability of this having occurred in all of the genomes with the genes of interest is low, considering that genes with functional relationships typically exhibit proximity in a genome, and fusions often happen between functionally associated genes (41). This filter criterium also gives strength to the identified cases, since it reduces false positives in the split component side that could arise from the presence of highly distributed and frequent domains in the genomic data set (e.g., flavin or iron-sulfur cluster domain). As an example, the biotin-dependent pyruvate carboxylase has its subunits fused in some bacteria (PycAB); however, split genes have lost operonic organization in some lineages (58). In archaea, the PycAB fusion is not identified, but instead, in *Methanocella*, PycA is found fused to biotin ligase (BirA/Bpl), as its gene is often colocalized with *pyc* genes. Using the approach of adding non-syntenic split sets if the syntenic sets are present, we recovered an additional 296 split proteins, including the Bpl and the PycA of *M. jannaschii* (59). In addition, Eukaryotes are not included in the analysis. Since the commonly accepted view is that eukaryotes evolved after the diversification of the two prokaryotic domains (60, 61) and, possibly having as host a lineage most closely related with the Asgard group (8, 62, 63), including eukaryotes would allow us to potentially identified events that happened in this domain, and not within Archaea, the focus of our study.

### Classification of protein fusion/fission families

The general lack of experimentally validated proteins for composite and split states prevents from using many classification algorithms. Therefore, a simple heuristic approach based on functional and genomic criteria was implemented to classify protein clusters as containing fusions or fissions. The syntenic split set frequency in the family was used for the initial separation between fission and fusion events (less than 0.1 for a fission, more than 0.9 for a fusion) (Fig. 2a). For high confidence fission assignments, besides a split frequency below 0.1, we relied on the presence of the respective composite protein in high-quality archaeal genomes and within bacterial lineages (see methods). KO model coverage (partial versus full) of the split proteins in comparison to the composite one was considered as well. Cases in which the split frequency was above 0.1 but the remaining criteria fulfilled were assigned as “high confidence” fission families. Clusters with a frequency above 0.1, where only some of the criteria were fulfilled, were assigned as probable fission. The presence of complete genomes, with full KO model coverage for the split proteins, and a limited distribution within bacteria were the criteria for a fusion assignment. Based on their presence/absence pattern, in combination with split frequency, the assignments of “high confidence” or probable fusion were given. As in fissions, cases in which, the split frequency was below 0.9 but the remaining criteria fulfilled were assigned as “high confidence” fusion families.

Often, proteins sharing a cluster can be both composite and split at the same time (Fig. 2b). Such overlap can be the result of concurrent fusion and fission processes or a series of fusion events that happened within that homologous family. In this case, if the split frequency is low, and split proteins are in low assembly level genomes (contig or scaffold), the protein cluster would be classified as probable fission. If within a cluster, a composite protein is itself part of a split set for a protein with more complex domain architecture also present in the cluster, then the cluster has the “fusion and fission” assignment. In the second largest “fusion and fission” cluster, besides “canonical” two domain peptide/nickel transport ATP-binding cassette (ABC) proteins and the gene products corresponding to a fusion of two peptide/nickel transport ATP-binding cassette (ABC) subunits from *Halobacteria*, also split products corresponding to the single ATP-binding subunit are found. Finally, when neither frequency nor any of the other criteria were supported, the cluster would remain unclassified.

As a result, 395 (28,8%) high confidence fissions and 289 (21,0%) fusions were identified. The remaining protein clusters were distributed among probable fission (20,4%) /fusion families (13.8%) or were a combination of both fusion and fission features in the same protein cluster (4.9%) with 11.1% remaining unclassified. The number of fusion/fission composite proteins correlates (r > 0.9) with the number of proteins per assembly, corresponding approximately to 10% of the total number of proteins per assembly. However, the correlation gets weaker in the case of fusions (r ~ 0.8), with fusion-derived composite proteins comprising ~1% of the total number of proteins in a genome (Fig. 2c through e). Regarding the split state, the number of sets highly correlates with the total number of proteins per assembly (Fig. S1). However, the correlation is lost for the high confidence fissions where only 119 assemblies contributed more than five syntenic split protein sets (Fig. S2). This high number of syntenic split proteins could be an indication of assembly fragmentation, particularly in the case of two *Thermoproteales* genomes. One of them, *Vulcanisaeta souniana* (IMG_2681813013) (64), has 148 out of 264 protein sets attributed to high confidence fission events, while on average, *Thermoproteales* assemblies only contain 56 split syntenic sets.

In some cases, the classification was difficult to assess, especially when homologous proteins were present in the same cluster or, when the split and composite state was inconsistent between archaeal and bacterial lineages (see Supplementary Information).

### Taxonomic distribution and functional annotation of fusion/fission families

**Fig 3:**
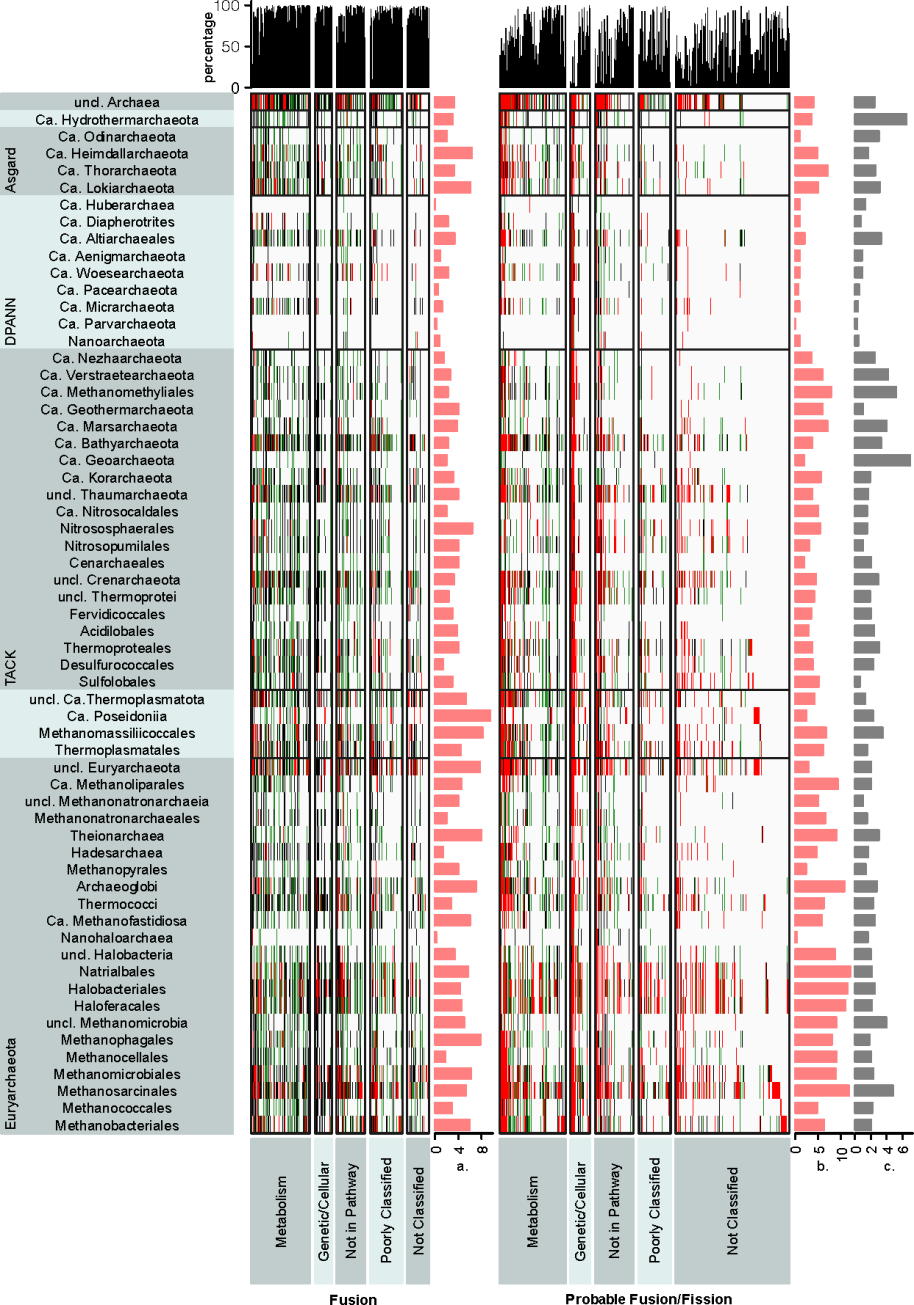
Taxonomic distribution of fusions across 1,678 archaeal assemblies. The taxonomic level, represented on the vertical axis, is grouped by order, phyla, or superphyla (indicated in bold). Protein clusters/families are represented on the horizontal axis, with functional category indication underneath. High-confidence fusions are represented on the left side, with probable fusions on the right side. Black indicates the presence of split syntenic sets, green of split sets (where syntenic representatives are absent), red of composite proteins, and white absence of any within the taxonomic rank. The top bar chart shows the percentage of split proteins over the total number of proteins per family. Singletons were excluded from the figure. On the right, the row annotation bar charts show (a) the average number of fusion events per genome (composite protein count); (b) the average number of probable fusion events per genome (composite protein count); and (c) the average number of probable fission events per genome (split protein count).

Except *DPANN* and *Ca*. Hydrothermoarchaeota lineages, fusions and fission proteins are widely spread across archaeal lineages (Fig. 3; Fig. S2; Table S5). High confidence fission protein clusters contain on average 12 times more proteins than high confidence fusion clusters. This apparent counterintuitive finding can be attributed to the occurrence of fission proteins within families that contain a higher number of composite proteins where only a minority of proteins are split into separate entities. In fact, fissions are more prevalent in protein families associated with housekeeping processes (e.g., informational processes) than with metabolic processes (see below). However, it can be observed that some lineages have a higher prevalence of protein families in which such events (fusion or fission) occurred. This applies to the halobacterial orders, *Methanosarcinales* and *Methanomicrobiales* in the case of fusions (Fig. 3) and *Methanosarcinales, Thermoproteales,* and *Thermoplasmatales* in the case of fissions (Fig. 3; Table S6). As the number of representatives per lineage is not uniform, we considered the distribution of families in the composite and split state on the genome level (Fig. 3; Fig. S3). For instance*, Methanosarcinales* and *Halobacteria* retain elevated numbers of fusion-fission events per genome (~180 families per genome, Fig. S3), but, on average, genomes of *Methanophagales* and *Ca*. Poseidoniia are more enriched in high-confidence fusions. The full representation of fusion/fission family diversity per lineage can be achieved with at most 75 assemblies, while for some lineages it takes only ~25 (Fig. S4). Performing an analogy with pangenomic analysis (65), it seems with regard to fusions, a closed “pan-fusion” behavior is observed. Several events of fission and fusion were identified in unclassified *Archaea* or unclassified *Euryarchaeota*. However, in MAGs with unclear taxonomic placement, the quality of the assembly can lead to technical artifacts in genome-wide applications.

**Fig 4:**
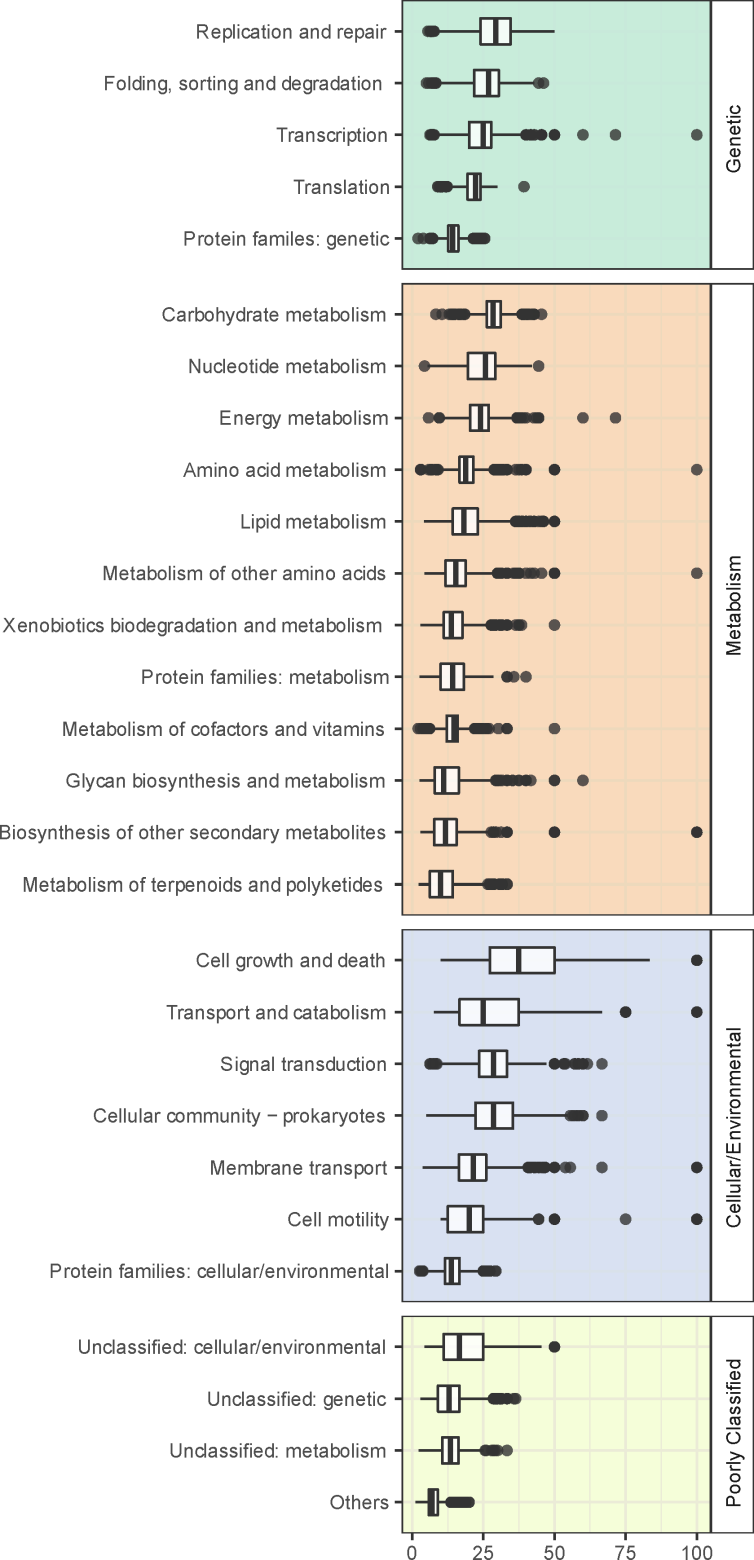
Relative abundance of fusion/fission proteins versus the total number of proteins identified per functional category (KEGG categories and KO annotations per assembly were utilized to calculate the ratio).

The functional annotations indicated that fissions tend to prevail in proteins related to genomic information processing and carbohydrate metabolism, while fusions are mainly found in quorum sensing, cell motility, chemotaxis, and poorly characterized proteins. (Fig. S2; Fig. 4). When compared to other categories, energy metabolism and amino acid biosynthesis categories are enriched in fission and fusion events (Fig. 4), both represented equally. Notably, a fusion of two or more proteins usually affects only the C- or N-terminus, with possible length reduction, however, does not have an impact on the active or binding sites (Fig. S5).

Functionally, the largest fusion/fission clusters were assigned to ABC transporters, accommodating ~8% of the total number of composite proteins. Among those, 34% are annotated as ABC-2 type, a largely uncharacterized family, universally present in Bacteria. Among the highly populated clusters, three others comprise sequences classified as ABC-transporters, specialized in transporting nickel/peptide, amino acids, and branched sugars, respectively. However, due to their high sequence homology, the functional affiliations between these clusters partially overlap. The lack of experimental validation regarding the specificity of ABC transporters does not allow us to apply the existing ABC-transporter structural classification (66) to the data set.

Apart from transporters, sequences from clusters universally distributed among this data set often belong to the oxidoreductases category. The remaining protein families with large taxonomic representation are affiliated to central carbon metabolism, DNA/RNA processing, or two-component system. Both taxonomical and functional affiliation of the clusters containing mapped split components indicate that these families were mainly retrieved due to scattered cases of fission. A closer inspection of these cases showed common trends regarding the low level of genomic assembly (scaffold or contig) and the lack (or low coverage level) of KO assignments for the unfused components, suggesting their retrieval due to a technical rather than biological fission. This once again indicates that the level of gene fragmentation or frameshifts is not always captured by standard genome quality assessment criteria.

Among archaeal lineages, *Methanosarcinales*, *Methanomicrobiales*, *Methanobacteriales*, and *Ca*. Poseidoniales contribute to approximately half of the lineage-specific protein clusters (162/347 clusters). Notably, functional categories such as O-Antigen and lipopolysaccharide biosynthesis proteins, glycosyltransferases, and transporters are highly represented in these identified protein families. A considerable fraction (~50% corresponding to 173 clusters) of the clusters consist of poorly characterized enzymes and proteins with unknown function.

When focusing on class-specific fusions, halobacterial proteins stand out, being present in 138 protein clusters (Fig. S6). However, no clear patterns of either fusion or fission or functional module enrichment are observed, apart from poorly classified signaling and cellular processes, to which 16 of these clusters are affiliated. In addition, approximately 50% of the clusters have no defined function according to the KEGG database. Within methanogens, 200 unique protein clusters containing fusion/fission proteins were identified, some of which are associated with energy metabolism, in particular methane metabolism.

The mapping of the identified archaeal composite proteins to bacterial assemblies revealed that although many of the protein families have homologs widely distributed across bacterial groups (Table S7), for 162 cases, no bacterial composite homologs were identified. If we considered a “minimum of two assemblies per lineage” rule, this number would increase to 228 non-singleton clusters (Fig. S7). These clusters correspond to unique archaeal composite proteins. These unique archaeal fusion/fission families belong to the genetic information processing (eight clusters) and metabolism (19 clusters) categories (Table S2). Within the metabolism category, archaeal-specific fusion clusters were found to have occurred among genes from folate, methane, and amino acid metabolism.

### Fusion/fissions events in archaeal metabolism

**Fig 5:**
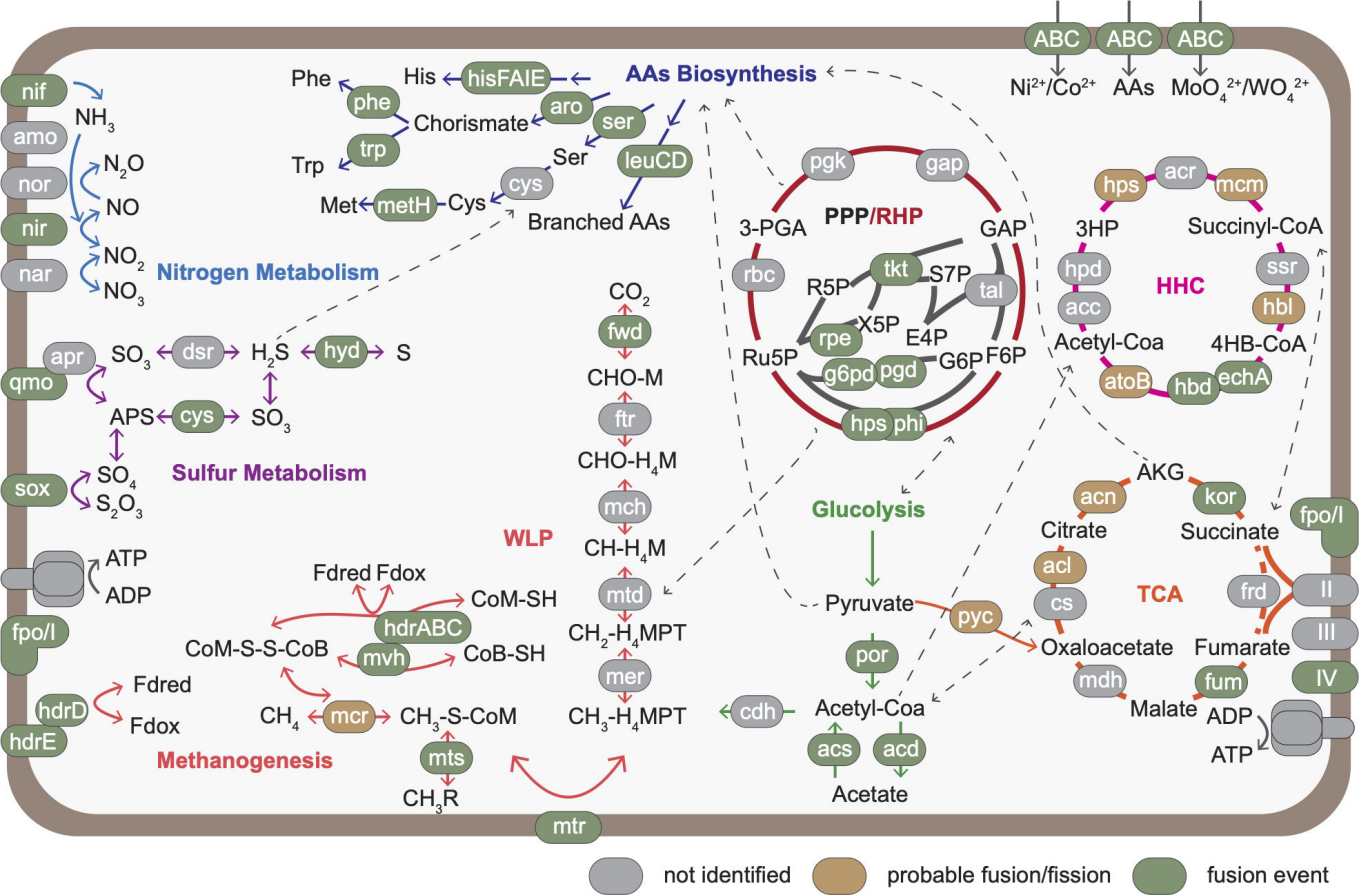
Fusion events in archaeal metabolism. The listed abbreviations do not occur in the text. Protein abbreviations. HHC: Acc, acetyl-CoA carboxylase; Hhps, hydroxypropionyl-coenzyme A synthetase; Acr, acryloyl-CoA reductase; Ssr, succinate semialdehyde reductase; Hbl, 4-hydroxybutyrate-CoA ligase; AtoB, acetyl-CoA acetyltransferase; Hpd, hydroxypropionyl-CoA dehydratase. PPP/RHP: Pgk, phosphoglycerate kinase; Gap, glyceraldehyde-3-phosphate dehydrogenase; Tal, transaldolase. TCA: Cs, citrate synthase; Frd, fumarate reductase. Respiratory chain: Nuo, NADH-quinone oxidoreductase; Qcr, quinol-cytochrome *c* reductase; Cyo, cytochrome-*c* oxidase. WLP: Fwd: formylmethanofuran dehydrogenase; Ftr, formylmethanofuran–tetrahydromethanopterin N-formyltransferase; mch, methenyltetrahydromethanopterin cyclohydrolase; Mtd, methylenetetrahydromethanopterin dehydrogenase; Mer, 5,10-methylenetetrahydromethanopterin reductase. Methanogenesis: Mcr, methyl-coenzyme M reductase; Mvh, F_420_-non-reducing hydrogenase; Fpo, F_420_H_2_: phenazine/quinone oxidoreductase. Sulfur metabolism: Sox, sulfur-oxidation system; Qmo, quinone oxidoreductase; Apr, adenosine 5′-phosphosulfate reductase; Dsr, dissimilatory sulfite reductase; Cys, sulfate assimilation enzymes. Nitrogen metabolism: Nar, nitrate reductase; Nir, nitrite reductase; Nor, nitric oxide reductase; Amo, ammonia monooxygenase; Nif, nitrogenase. AAs biosynthesis proteins: HisFAIE, histidine; Aro, chorismate; Cys, cysteine; Phe, phenylalanine; Trp, tryptophane; MetH, methyltetrahydrofolate-homocysteine methyltransferase; LeuCD, leucine; Ser, serine. Others: Por, pyruvate-ferredoxin/flavodoxin oxidoreductase; Cdh, carbon-monoxide dehydrogenase; Acs, acetyl-CoA synthetase; Pyc: pyruvate carboxylase; Compound abbreviations. HHC: 3HP, 3-hydroxypropionate; 4HB, 4-hydroxybutyrate. PPP/RHP: Ru5P, ribulose 5-phosphate; 3-PGA, 3-Phospho-D-glycerate; GAP, glyceraldehyde 3-phosphate; F6P, fructose-6-phosphate; R5P, ribose-5-phosphate; X5P, D-xylulose 5-phosphate; S7P, sedoheptulose 7-phosphate; E4P, erythrose 4-phosphate; G6P, glucose-6-phosphate. TCA: AGK, alpha-ketoglutarate; WLP: MF, methanofuran; H4MPT, tetrahydromethanopterin; Fd, ferredoxin; Sulfur metabolism: AAs biosynthesis: His, histidine; Ser, serine; Cys, cysteine; Phe, phenylalanine; Trp, tryptophan; Met, methionine.

The impact of fusion and fission events in archaeal metabolism is represented in Fig. 5 and will be discussed in detail below.

Within carbohydrate metabolism, both fission and fusion proteins were identified (14 fusions, 47 fissions, and 18 probable fusions/fissions). Notably, while in glycolysis 9 fissions were identified, carbon fixation and methane metabolism (assigned to Energy metabolism) show a more balanced distribution of both fusion and fission events (23 fusions and 13 fissions, in total, with “fusion and fission” families counting for both states) (Fig. 5). Within the tricarboxylic acid cycle (TCA), several fusion events were identified in both reductive and oxidative versions. For instance, a fusion was observed in the dihydrolipoamide dehydrogenase (LpdA, cluster 14_1), part of the aerobic pyruvate dehydrogenase complex, responsible for converting pyruvate to acetyl-CoA (67). In halophilic archaea, some LpdA proteins are fused to a biotin-dependent domain (cluster 14_1.2). On the other hand, anaerobic archaea use the alternative enzyme pyruvate oxidoreductase (Por) for the conversion of pyruvate to acetyl-CoA (68). A fusion between the alpha (PorA) and beta subunit (PorB) was identified in nine genomes, mainly in *Ca*. Bathyarchaeota and *Ca*. Korarchaeota (cluster 45.2). The enzyme aconitase A (AcnA) (69), responsible for converting citrate to isocitrate, can be the result of a fusion between two subunits from a methanogenic homocitrate aconitase (70) or subunits from the widely spread isopropylmalate dehydratase involved in leucine biosynthesis (70) (cluster 139). Two fusion events, involving 2-oxoglutarate oxidoreductase Kor subunits, a complex responsible for converting oxoglutarate into succinyl (68), were identified: one between *korA* and *korC* (cluster 75.1) genes, and another between *korB* and *korC* genes (cluster 368). Fusions tend to occur between closely related genes, and for this enzyme, there is a high conservation of synteny across the archaeal phyla. Moreover, the split components of this family include the experimentally validated KorB and KorC proteins from *M. marburgensis* (68), corroborating our prediction. In the case of fumarate hydratase (Fum) class 1, the subunits were identified as fused in most bacteria but in the split state in the majority of Archaea (71). Exceptions include two *Desulfurococcales* and two *ANME* assemblies, in which the fused version was identified (cluster 926). The subunits alpha (SucC) and beta (SucD) from succinyl-CoA synthetase, an enzyme that converts succinyl to succinate (72), were found in the split syntenic configuration in four and two assemblies, respectively (cluster 120). The *sucD* gene is also involved in a probable fusion event with a citrate synthase protein, forming the composite protein ATP citrate (pro-S)-lyase (*Acl*) (73, 74) which is found in some *Methanosarcinales* species (cluster 120). This enzyme is a part of the reductive TCA, converting citrate to acetyl-CoA, and was previously described in the split state in *Aquificae (*74) (cluster 547). Fissions were also identified in the flavoprotein subunit of succinate dehydrogenase (SdhA) (six assemblies, cluster 127_1) and between the two domains of malate dehydrogenase (eight split proteins, four assemblies, cluster 142). While in the first case, our analysis favors these to be the result of assembly artifacts (technical fission), the second represents a candidate for further experimental investigations.

The oxidative phase of the pentose phosphate pathway (PPP) is not widespread among archaea, and within this pathway, a single fusion event occurring between glucose-6-phosphate 1-dehydrogenase (G6pd) and 6-phosphogluconate dehydrogenase (Pgd) enzymes was identified in *Diapherotrites (DPANN* phylum, cluster 1367, singleton). Interestingly, the fused form is also present in 60 bacterial candidate phyla assemblies. By contrast, within the biosynthesis of ribose from fructose (the dominant route in Archaea), the pipeline identified the experimentally validated bifunctional enzyme (Hps-Phi) (55, 75), which is the result of a fusion between 3-hexulose-6-phosphate synthase (Hps) and 6-phospho-3-hexuloisomerase (Phi) (cluster 220, Fig. S5). While in bacteria, Hps and Phi were identified as two separate proteins, the distribution pattern in archaea suggests that it is likely a result of an archaeal fusion event (76). Fusions were also identified for proteins from the non-oxidative branch of the pentose phosphate pathway (nPPP). Among them was the three-domain bacterial transketolase (Tkt) found as a split protein pair in 441 archaeal assemblies (cluster 341.2). In a few representatives from the *DPANN* superphylum and *Ca*. Thorarchaeota, the protein was identified in the fused state. Notably, in *Thaumarchaeota* (73 out of 98 assemblies), the N-*terminus* of Tkt is fused to a ribulose-phosphate 3-epimerase (Rpe) enzyme (cluster 341.1). This fusion event suggests the potential presence of a complete functional nPPP in *Thaumarchaeota*, differentiating it from the typical pathway configuration found in most other archaeal lineages, as previously suggested (77–79). The identification of composed and split proteins suggests that the non-oxidative branch of the pentose phosphate pathway is more widely distributed in archaea (77–79). This indicates a greater prevalence and functional significance of nPPP in various archaeal lineages, contributing to a more comprehensive understanding of carbohydrate metabolism in these organisms.

Regarding the reductive hexulose-phosphate (RHP) pathway, several fissions of multidomain proteins have been observed, including the large chain of ribulose-1,5-bisphosphate carboxylase (RuBisCo/Rbc, cluster 236). However, no fusions were detected in this pathway. On the contrary, both fusion and fission events were observed in the 3-hydroxypropionate/4-hydroxybutyrate (3HP/4HB) carbon fixation cycle, a widely utilized pathway by diverse, mostly non-methanogenic archaeal lineages (80, 81). The pathway involves a series of carboxylation and reduction events and reuses the same domain architectures in different enzymes. Multiple enzymes of the 3HP/4HB cycle were found to be part of the same fusion/fission cluster, which also contain homologs involved in carbohydrate and fatty acid metabolism. In ammonia-oxidizing archaea (AOA), fusion events promoted the divergence and functional adaptation of the homologous enzymes, 3-hydroxypropionyl-CoA synthetase (Hps) and 4-hydroxybutyrate-CoA ligase (Hbl) (81). While the Hps enzyme, besides the shared domain with Hbl, contains an ATP-grasp domain fused to its N-*terminus*, the Hbl enzyme has the ATP-grasp domain at its C-*terminus*. Both Hps and Hbl enzymes cluster with acetate-CoA ligase (AcdAB) enzymes, which catalyze a similar CoA-dependent reaction (81, 82) (cluster 20). The corresponding split versions of the three enzymes were found in several extremophilic lineages, including *Desulfurococcales* (17/30) and *Thermoproteales* (24/35). In *Sulfolobales,* this enzyme is absent, since the organisms use alternative enzymes for carbon fixation (83). Phylogenetic reconstructions of this extended family could give insights and aid in the clarification of the precise order of these events. An additional fusion between 3-hydroxybutyryl-CoA dehydrogenase (Hbd) and crotonyl(enoyl)-CoA hydratase (EchA), two enzymes involved in consecutive steps of the pathway (84) was also identified (cluster 65, Fig. S5). The EchA-Hbd fusion is widely distributed in the *TACK* superphyla (186/425), the majority of halophiles (374/412), and in *Archaeoglobales* (13). On the other hand, split versions of these enzymes occur in *Sulfolobales* (~97%), ~50% of *Halobacteria,* and 85% of the AOA assemblies analyzed here. In the unfused Hbd proteins from AOA, a duplication of the C-*terminus* domain is conserved in all of the fused components identified (cluster 65). In the halobacterial version of Hbd, however, this duplication is absent (cluster 65.3). In addition, a fusion between acetyl-CoA C-acetyltransferase, an enzyme responsible for acetyl-CoA regeneration (81, 84), and an uncharacterized conserved protein was identified in two *Thermoplasmatales* genomes (cluster 17.2). The corresponding split pairs tend to be syntenic in 12 phyla, predominantly from *TACK* and *Asgard* supergroups.

In the context of methane metabolism, protein families display a balanced rate of fused and unfused components (Fig. 5). Many reactions of the pathway rely on multi-subunit complexes (85), and we observe multiple fusion combinations per protein complex. The initial step of methanogenesis, where CO_2_ reduction is initiated, is catalyzed by formylmethanofuran dehydrogenase (Fwd/Fmd) (86, 87) which contains between 6 and 8 subunits. In 16 *Methanomicrobiales* assemblies and 14 *TACK* assemblies, fusions of subunits B and D were identified (cluster 525). In addition, in 3 assemblies of *Methanosaeta*, a fusion between Fmd subunit E and a transcriptional regulator is present (cluster 663.2–3). In some *Methanosarcinales* (4) and *Archaeoglobales* (3), FmdE is fused with a tRNA methyltransferase domain (cluster 663.1). The identified fusions between the only *fmdE* gene copy and a tRNA methyltransferases domain might indicate the peripherical role of this subunit within the complex, pointing to a possible regulatory function. In fact, in the recently obtained structure of the complex (87), the FmdE subunit is not observed, which can further corroborate this idea. Other events can be found in Table S2.

Three fusion events were identified between the subunits of tetrahydromethanopterin S-methyltransferase (Mtr), a key enzyme involved in methyl-coenzyme M formation (88). These include a singular fusion of subunit A to B in unclassified *Thaumarchaeota* (cluster 797.1), between subunit A and H in unclassified Archaea and between subunit A and F in 12 *Methanococcales* and 2 *Methanocellales* assemblies (cluster 797.2).

The Methyl-coenzyme M reductase (Mcr) enzyme is responsible for catalyzing the reduction of methyl group to methane through a reaction between coenzyme M and B (89). In the assembly of *Methanoculleus marisnigri,* its subunit A is split into two proteins (cluster 306.1). This finding is particularly important since the subunit is widely used for phylogenetic reconstructions (90), and proper recognition of fission events is crucial for accurate analyses.

Finally, within the heterodisulfide reductase complexes (HdrABC and the HdrED present in cytochrome containing methanogens) that catalyze the reduction of coenzyme B (CoB) and M (CoM) (91, 92), several fusions between their subunits were identified (Table S2; Fig. S5). These are widely distributed across taxa, can involve more than two subunits, and may include fusion of duplications, as seen in the alpha subunit (HdrA). For instance, *Ca*. Thorarchaeota (16/18 assemblies) and *Ca*. Lokiachaeota (9/10 assemblies) have multiple copies of the *hdrA* gene per assembly (cluster 77). In all of these lineages, at least one of these HdrA proteins is the short version of the enzyme, having on an average of 650 amino acids. In addition, proteins resulting from a fusion of two HdrA domains are further fused with an F420-non-reducing hydrogenase iron-sulfur subunit D (mvhD) that is often attached to its C terminus (17/54 duplicated HdrA fusions) (cluster 77). These longer versions of HdrA proteins were identified to be in up to six copies per assembly. Except *Thaumarchaeota* where only in two assemblies four HdrA fusions were detected, a high number of fused HdrA domains are found within the *TACK* phyla. Methanogens, however, have only one or two HdrA copies in the cluster, most of them corresponding to the “canonical” shorter form or instead, fused with the mvhD domain.

Fusions are also observed in the enzymes responsible for the biosynthesis of cofactors related to methanogenesis. For instance, fusions were identified between sulfopyruvate decarboxylase subunits (ComE and ComD), an enzyme involved in the biosynthesis of coenzyme M (93) (cluster 348). This fusion was identified in 175 assemblies from 16 taxa, excluding uncharacterized *Archaea* and uncharacterized *Euryarchaeota*. These findings highlight the diversity and evolutionary adaptations in carbon fixation pathways and its associated cofactor biosynthesis enzymes among archaeal lineages, shedding light on the complex metabolic strategies employed by different archaea in response to their environmental niches, in particular in terms of carbon metabolism.

### Nitrogen and sulfur metabolism

Among archaeal fusion/fission protein families, there are notable representatives involved in nitrogen metabolism (seven clusters) and sulfur metabolism (11 clusters). However, it is worth mentioning that these fusion events predominantly occur within assimilatory routes, being scarce in dissimilatory ones. Archaeal fusion/fission protein families are spread across diverse aspects of the nitrogen cycle, primarily in assimilatory nitrogen reduction, denitrification, and nitrogen fixation (11). However, it is important to note that the majority of these protein clusters are the result of fission events. One way to assimilate nitrogen is the uptake of nitrate and its conversion to ammonia via nitrite (11). The subsequent nitrite reduction step is catalyzed by the ferredoxin-nitrite reductase (NirA) (94) which is homologous to the sulfite reductase (Sir) (94), involved in sulfur metabolism. NirA is a four-domain protein, arranged in an A-B-A-B manner. NirA is widely present in *Halobacteria* where 19 out of the 529 identified proteins are fused to a rhodanase domain (cluster 195.2). Rhodaneses are sulfur carriers involved in sulfur metabolism (95), and these halobacterial composite proteins might perform a role in sulfur metabolism. The four-domain NirA itself might be the result of a fusion between the two domain proteins (A-B), identified in *Methanosarcinales*. Sir and NirA (94) are found in the same cluster. Both could have emerged from the fusion of already duplicated proteins before their functional specialization.

A fusion event involving a NO-forming, copper-containing nitrite reductase nirK (96, 97), and a plastocyanin, is present in four *Halobacteria* (cluster 505). In the majority of *Halobacteria*, however, the NirK and plastocyanin proteins are found in the split version, conserving synteny. A similar copper-containing protein architecture with a plastocyanin extension is observed across *Thaumarchaeota (18*), including AOA, where NirK is hypothesized to be involved in N_2_O production (98) (cluster 505). In addition, one high-confidence fusion between NifH and NifD/E proteins in *Methanomassiliicoccales* was identified, while in *Methanosarcinales* and *Methanomicrobiales* both composite and split forms are found (cluster 443).

In sulfur metabolism, only a few fusion/fission events were identified, with fission events prevailing, especially in the assimilatory reduction pathway. Among the proteins involved in the assimilatory pathway, three distinct protein clusters were identified. The first corresponds to sulfate adenylyltransferase (Sat), which catalyzes the initial activation step of sulfate to adenosine 5′-phosphosulfate(APS) (99). The Sat cluster was retrieved due to a single fission event in the *Vulcanisaeta sounina* IMG assembly (cluster 407.1). A single case of Sat protein fusion with histidine phosphatase was also registered in a *Sulfolobus* genome (cluster 407.2). The second cluster corresponds to phosphoadenosine 5′-phosphosulfate reductase (cysH), which is responsible for the third step in the pathway (100) (cluster 536). This cluster is exclusively composed of *Methanomicrobiales* proteins. Interestingly, the domain architectures suggest a fusion of CysH with a cysteine desulfurase (SufS), an enzyme involved in amino acid biosynthesis (101, 102) (cluster 536). However, only in four assemblies, the unfused gene sets were observed, hindering a clear fusion/fission classification. In addition, a fusion between *cysH* and the threonine synthase (*thrC*) gene is observed in three *Thermoplasmata* assemblies (cluster 1057).

In contrast to the assimilatory reduction pathway, no fusion or fission events were identified in enzymes responsible for the conversion of sulfite to sulfide in the dissimilatory sulfate/sulfite reductive pathway. Only in the case of *V. souniana,* the adenylylsulfate reductase(Apr) that catalyzes the reduction of APS to sulfite (103) was observed as a split set. This was previously reported and currently, it is not clear if the Dsr-cascade is operational or not in this organism (23). Of note, a fusion of the siroheme domain also present in DsrAB and AsrC proteins with the F_420_ domain as seen in the case of the F_420_-dependent sulfite reductase, Fsr, was observed. This protein is present in methanogens and has a higher identity to the anaerobic sulfide reductase (AsrC) rather than with DsrAB proteins. AsrC, as well as Fsr, has an additional iron-sulfur cluster (57, 104, 105), and it is proposed that Fsr originated from a fusion between a siroheme-domain containing AsrC ancestral domain and the FrhB domain (56).

Lastly, a fusion between the sulfur hydrogenase beta and gamma subunits, which are involved in sulfur/sulfide reduction, was observed. This composite protein has so far only identified in 11 archaeal assemblies, mainly in *Thermoplasmatales, Ca*. Bathyarchaeota, as well as several bacteria.

### Complexes from mitochondrial-like respiratory chains

The mitochondrial electron transport chain is characterized by the presence of four complexes (I to IV) and an ATP synthase. Each one of these complexes has homologues within the prokaryotic domain, namely in Archaea. Complex I is part of group 4 of membrane-bound [NiFe] hydrogenases and within Archaea, due to the inexistence of the electron input module NuoEFG, denominated as Fpo (F_420_H_2_:phenazine oxidoreductase) (106), or Fqo, F_420_H_2_ quinone oxidoreductase (107). A fusion between the subunits B and C of this complex was identified in 47 assemblies with diverse taxonomic affiliations (cluster 482), with 10 of these cases being further fused to subunit D (cluster 482). Importantly, in the initial group (fqoBC), the protein from *Archaeoglobus fulgidus* is present, as previously suggested to be a fusion of those subunits (107). Regarding the terminal heme-copper oxygen reductase (Complex IV), the fusion between subunits I and III was identified (cluster 251), in which, the experimentally characterized *S. acidocaldarius* protein is present. The possibly identified fission event regarding complex II or succinate dehydrogenase was already discussed above (TCA cycle).

### Amino acid metabolism

The majority of fusion events related to amino acid metabolism are present in the biosynthesis of histidine and aromatic amino acids pathways. The initial steps of histidine biosynthesis are catalyzed by the bifunctional fusion protein phosphoribosyl-AMP cyclohydrolase/phosphoribosyl-ATP pyrophosphohydrolase (HisIE) (108). While initially thought to be widely distributed only in *Thermococcales* and *Thermoplasmatales* (109), this fused form has now been found in some novel methanogenic lineages, including *Methanomassilococcales*, *Methanofastidiosa*, and *Methanomethyliales* (cluster 384). On the other hand, split syntenic pairs of HisIE are present in *Thermoproteales*, *Ca*. Bathyarchaeaota, *Ca*. Hadesarchaea, and *Desulforococcales* (cluster 384). The emerging distribution of HisIE suggests that the fusion might have originated within the archaeal clade itself, providing a possible alternative to the previously suggested horizontal gene transfer from bacterial partners (109). Another intriguing fusion event involves two subunits of imidazole glycerol phosphate synthase (HisH and HisF), found fused mostly in eukaryotic species (110). The fused HisHF is present in 2 *Archaeoglobales* species and has approximately 70% identity to HisHF from sulfate-reducing bacteria (cluster 787.3). However, the identified HisF may also function as a phosphoribosylformimino-5-aminoimidazole carboxamide ribotide isomerase (HisA) since they are homologous and have evolved from an ancient duplication (109). Moreover, HisA/F fusions with HisIE (phosphoribosyl-AMP cyclohydrolase/phosphoribosyl-ATP pyrophosphohydrolase) have been identified in four metagenomic assemblies, scarcely distributed across *DPANN* and unclassified Archaea (cluster 787.1). Interestingly, the bifunctional imidazoleglycerol-phosphate dehydratase/histidinol-phosphatase (HisB) (111) and the fusion of HisIE with histidinol dehydrogenase (HisD) (112), present in Bacteria and Eukarya (109*),* were not identified in the Archaea domain.

In the biosynthesis of chorismate, a key precursor of aromatic amino acids, several composite proteins have been identified (Fig. 5). A fusion between 3-dehydroquinate dehydratase (*aroD*) and shikimate dehydrogenase (*aroE*) genes, first discovered in plants (113), was also identified in some archaea, including 8 *Methanofastidiosa* and 10 *Methanomassiliicoccales* assemblies, as well as in two bacterial phyla (198 assemblies of *Planctomycetes* and 88 assemblies of *Acidobacteria)* (cluster 359.1). A fusion involving *aroE* and the shikimate kinase *aroK* (114) genes has been identified in *Methanomicrobiales* (42/55), one unclassified *Methanomicrobia,* and two bacterial assemblies (cluster 319).

In the tryptophan biosynthesis pathway, the composite protein anthranilate synthase/phosphoribosyltransferase (TrpGD) catalyzes the initial steps of the pathways in a range of bacteria (115) (cluster 373). Interestingly, *trpGD* was found fused to a nitric oxide reductase gene (*nor*) in one *Methanocella* genome, indicating a unique evolutionary event in this archaeal lineage or a result from interdomain LGT (cluster 373.2). A fusion between *trfD* and *trfC* genes was also identified in some organisms (cluster 940). In addition, a fusion between phosphoribosylanthranilate isomerase (*trpF*) and tryptophan synthase beta chain (*trpB*) (116), although rare, has been identified in both bacterial and archaeal domains (cluster 830.1, single fusion protein trpAB in 830.2). Finally, in two assemblies, a fusion between *trpF* and *trpC* was identified (cluster 1136).

In the biosynthesis of phenylalanine, a fusion between chorismate mutase and prephenate dehydratase (PheA) (117) is common among bacteria (present in 9,768 bacterial assemblies). However, within archaea, this fusion only occurs in a few representatives of the *DPANN* and *TACK* superphyla (cluster 245.2). Moreover, in *Archaeoglobus* and *Ca*. Huberarchaea, a second step fusion has been identified, where chorismate mutase/prephenate dehydratase is further fused to a prephenate dehydrogenase (118), an enzyme involved in the first step of tyrosine biosynthesis (cluster 693). This multi-fusion event appears to be unique to these two archaeal lineages.

### Cofactor biosynthesis

Cofactor biosynthesis plays a vital role in cellular metabolism, as these essential molecules can be recycled and used multiple times by enzymes with divergent functions and evolutionary histories. Thus, understanding the evolution of cofactor biosynthesis enzymes can provide valuable insights into the early evolution of life. Through comprehensive screening, numerous fusion events have been identified in various biosynthesis pathways, including those for thiamin, biotin, riboflavin, tetrahydrofolate, molybdopterin, and heme biosynthesis. Below, we give the details of the latter two pathways.

Molybdopterin biosynthesis is a crucial pathway involved in the synthesis of a cofactor that binds to a broad range of oxidoreductases (119). In this biosynthetic process, several fusion and fission events have been identified for its enzymes. Notable fusion events involve the cyclic pyranopterin monophosphate synthase MoaC (120), which has been independently fused on multiple occasions with itself (cluster 625.1) as well as to enzymes of two downstream biosynthesis reactions, namely MoaE and MoaB (120) (cluster 625.2 and 765, respectively). These fusions are not limited to specific taxonomic groups and could be a result of frameshifts in the conserved syntenic blocks. Interestingly, no fusion between MoaC and GTP 3′,8-cyclase (MoaA) (121), an enzyme that initializes the pathway, was identified. On the other hand, fusions involving MoaD, a sulfur-carrier protein (120, 121), are lineage specific. For example, the MoaD-MoaE fusion (subunits of molybdopterin synthase) has been identified in (18 out of 35) *Thermoproteales* and (5 out of 30) *Desulforococcales*, as well as in a small fraction of bacterial genomes (775/33957) (cluster 419.1). However, the protein was proposed to be non-functional in its fused form, as the C-*terminus* of MoaD needs to remain open to bind the sulfur group, requiring post-translation cleavage (122). The fusion between MoaD and the molybdopterin-synthase adenylyltransferase (MoeB), the protein responsible for the MoaD adenylation (123), is present in *Thaumarchaeota* (61/98) and unclassified *Crenarchaeota* (4/246) and two other assemblies (cluster 356.1). In bacteria, this fusion is limited to *Ca*. Rokubacteria and *Ca*. division NC10, both of which are hypothesized to be nitrite reducers (124). Moreover, the *moaD* fusions with *moeBR* gene, where *moeB* is fused with a rhodonase domain (cluster 356.3, 14 assemblies) and *moeBR* (cluster 356.2, 25 assemblies) were identified mainly in unclassified *Euryarchaeota* and *Ca*. Poseidoniales. This suggests the possibility of a gene transfer event from *Acidobacteria* to Archaea, given the relatively high identity (over 55%) between the proteins from these two groups. The last enzyme in the molybdopterin biosynthesis pathway, MoeA, is responsible for catalyzing the insertion of molybdenum into molybdopterin (125). The protein appears to have fissions(cluster 27.1) as well as periplasmic domain extension (cluster 27.2), classified as fission with both cluster subgroups lacking split components. The taxonomic distribution of the fused and split enzymes is not uniform in Archaea, indicating a complex evolutionary history for molybdopterin biosynthesis across different taxa.

Heme is a crucial iron-containing porphyrin cofactor involved in electron transfer, essential for both aerobic and anaerobic respiration. Prokaryotes utilize three distinct pathways for heme biosynthesis, converging at uroporphyrinogen-3 as their last common intermediate (126). Fission and fusion events are common occurrences in the enzymes catalyzing early reactions (HemABLCD) of the pathways from glutamate to uroporphyrinogen-3. Our analysis revealed the existence of several fusion proteins in this pathway. One such fusion event was found between glutamyl-tRNA reductase (HemA) and porphobilinogen synthase (HemB), in a single *Sulfolobus* assembly. In addition, two fusion events were identified between hydroxymethylbilane synthase (HemC) and the composite protein uroporphyrinogen III methyltransferase/synthase (CobA-HemD) in *Euryarchaeota* (cluster 192.3). CobA-HemD (127) is a fusion protein in itself, where CobA is responsible for catalyzing precorrin-2 biosynthesis, representing the first divergent reaction in the alternative heme biosynthesis pathway. This fusion protein was initially characterized in sulfur-dependent bacteria (127) and based on our screening results, it is also present in *Actinobacteria* (3448/5525), *Firmicutes* (1974/5564), and around 100 ammonia-oxidizing bacteria (cluster 192). However, although the synteny of the split proteins was universally conserved across other archaeal lineages (cluster 605.1) within Archaea, the fused CobA-HemD proteins were mainly identified in the genus *Methanothrix* and *Ca*. Methanofastidiosa.

Fission and fusion events were also identified in the alternative heme biosynthesis pathway, where siroheme serves as an intermediate (126). In 14 lineages of *TACK* and the majority of *Euryarchaeota*, siroheme decarboxylase genes *ahbA* and *ahbB* were found fused, while they remained split in the majority of *Methanosarcinales*, as previously reported (128*),* and also in *Methanomicrobiales* and *Methanonatronarchaeales* genomes (cluster 230). Interestingly, a fusion between *ahbAB* and *ahbD* (AdoMet-dependent heme synthase) genes was identified in five genomic assemblies affiliated with *Methanomicrobia* (cluster 36.2). These fusion and fission events in the heme biosynthesis pathway illustrate the dynamic nature of enzyme evolution and how organisms can utilize different strategies to adapt and regulate their cellular processes, particularly in the context of synthesizing essential cofactors like heme.

## DISCUSSION

The identification of fusions and fissions in prokaryotic genomes has raised several technical and biological questions. We observed that the number of identified fissions increases with the growing number of incomplete genomes, leading to challenges in elucidating biological meaning from the noise in the data. Thus, we speculate that many of our identified fissions and fusions might be due to the incorrect assembly of public metagenomic records. Here, we chose to include records with heterogenous data quality to explore their impact in this and other analyses.

The distribution of bacterial and archaeal protein families often shows polar patterns, which can be attributed to various factors like the absence of certain proteins, interdomain horizontal gene transfers, poor alignment coverage of split pairs from distant lineages, or misidentification of protein domains. Efforts have been made to address some of these issues through clan mapping, but many protein domain models need to be updated with diversified archaeal sequences. Even the currently available archaeal clusters of orthologue proteins are based on a reduced number of archaeal lineages and sometimes contain multiple paralogs within a group (129).

This screening also revealed a lack of functional characterization in the archaeal domain. In some cases, poorly characterized proteins are fused with experimentally validated ones, providing insights into their potential functions. However, in many instances, both split proteins have unknown functions, making functional deductions challenging. To fill these knowledge gaps, more experimental characterizations, especially in what concerns protein from the archaeal domain need to be performed.

The set of candidate fusion families was constructed by combining protein domain and full sequence similarity between composite and split proteins. Additional classification was applied to distinguish between fusion and fission events, with fissions typically having a wider taxonomic distribution and being involved in genetic information processing, while fusions were more commonly associated with taxonomically restricted metabolic processes. These results highlighted unique archaeal fusions and revealed underestimated the capacity for energy metabolism in certain lineages (carbon fixation, e.g., cluster 65), as well as the importance of fusion processes in halobacterial signaling and cellular processes (two-component system/chemotaxis proteins in multiple clusters; NO signaling e.g., cluster 835; flagellins, e.g., cluster 575, cluster 89).

In conclusion, the identification of fusion and fission events in prokaryotic genomes has shed light on various aspects of microbial physiology and evolution. The study has underlined the need for integrating new steps into genome assembly quality assessment pipelines to improve the accuracy of fusion identification. Overall, the findings contribute to a deeper understanding of the functional diversity and metabolic capabilities of prokaryotic lineages, particularly Archaea, and highlight the importance of experimental validation to fill gaps in our knowledge.

## MATERIALS AND METHODS

### Data acquisition

A total of 2,693 archaeal assembly records were obtained from NCBI Archaea (Jan. 2020). In addition, 1,000 publicly available genomes of Archaea were retrieved from IMG (January 2020). Genome completeness and redundancy (contamination) levels were estimated on *faa files containing the annotated protein sequences using the set of archaeal marker protein HMMs (130). Both estimates were used as criteria for the data set filtering. The genome redundancy cutoff was set to 10%, while the completeness cutoff was optimized according to the taxonomic affiliation, to account for organisms known to have reduced genomes. In cases where strains were represented by multiple assemblies, the assembly level (complete genome, scaffold, etc.), completeness, and redundancy were used to select a single reference assembly. To reduce the total number of genomes, bacterial genomes were further filtered for cases of overrepresented species. In total, 1,678 archaeal and 33,957 bacterial genomes, downloaded from NCBI in January 2020 and November 2019, respectively, were used in this analysis.

### Fusion/fission family identification

Proteins from the archaeal genome data set were searched for protein domains (pfams) against Pfam32.0 (131) using HMMER3 (132). The HMMER3 output was processed with dPUC2 (133) to produce sets of protein domains with the highest pairwise directional probabilities. Next, the archaeal protein domain architectures were matched against each other to construct the preliminary set of fission/fusion protein families. When possible, and to expand the fusion protein search space, pfams were mapped to their respective clans, which contain two or more homologous families (134). For the assignments, the multidomain architecture of a single protein (composite protein) had to match the sum of the domains of the split protein pairs/sets, and, in the case of multidomain split components, also the order of domains had to be retained. Finally, among the genes encoding candidate unfused proteins at least one set should be genomically colocalized, in this case, a maximum of five genes apart (Fig. 1). The last condition has been derived from the operonic organization of prokaryotic genomes making the fusion between colocalized genes more probable. Candidate fusion proteins with identical domain architectures were grouped into preliminary fusion families, together with the corresponding subsets of unfused components.

### Similarity searches

The preliminary families were aligned with each other using DIAMOND blastp (38). To remain as the part of fusion family, candidate proteins had to fulfill several criteria. First, pairwise alignment between composite and split protein should have a sequence identity of more than 25% and an E-value less than 1e^−9^. The overlapping coverage between the candidate fusion protein and the potential split candidate should cover at least 60% of the latter. Neither of the proteins in the set should overlap with each other by more than 100 amino acids in the alignment to the composite subject. Moreover, at least 60% of a candidate protein sequence had to be covered by the split proteins. Finally, a fusion/fission family had to have at least one composite protein and one set of split proteins encoded by syntenic genes passing all listed filtering criteria (Fig. 1). When genes encoding the split proteins are not colocalized but have the same domain combination and cover the same composite protein as the ones in synteny, they were added to the split side of the fusion/fission family.

### MCL clustering and functional annotation

The composite proteins from predicted fusion/fission families were subjected to global alignment using the Needleman-Wunsch algorithm and clustered using the Markov algorithm (MCL) (135) with the inflation parameter set to 1.2. Inflation of 1.2 results in lower cluster granularity which can potentially lead to poorly related paralogues sequences being attributed to the same cluster. To split paralogs into orthologous groups, the clusters with more than 10 members were further subclustered at 1.6 inflation. If resulting subclusters demonstrated clear separation by the combination of existing functional annotations or/and sequence length while keeping maximum inter-cluster identities below 40% then they were kept, otherwise, it remained at 1.2. Apart from existing PFAM annotations, the cluster proteins were annotated using KofamScan, Kyoto Encyclopedia of Genes and Genomes (KEGG) (136) based software to retrieve orthology (KO) mappings. For KO models with no threshold to be considered, they had to be the protein’s best hit and with an E-value below 1e^−10^. The EggNOG database tool, eggNOG-mapper, was used to retrieve Clusters of Orthologous Groups of proteins (COGs), including arCOGs (137, 138). Finally, the transporter classification database (TCDB) (139) was used to refine transporter assignments.

### Bacterial mapping

The bacterial proteins were searched for protein domains with the same combination of tools as archaeal proteins. The proteins with the same composite domain architecture were annotated with KofamScan and aligned to the archaeal genomes the fusion/fission proteins came from using DIAMOND blastp. The output was filtered for the best one-directional hit. If the best hit was the predicted archaeal composite protein and both bacterial and archaeal proteins had identical domain architectures, the bacterial protein was mapped to the mcl cluster of the archaeal fusion protein. If the domain architectures differed, then it was counted separately. For each candidate, the bacterial protein with multiple best hits and the hit with the highest score per identical and non-identical domain architecture were picked to represent the final affiliation to a fusion/fission protein family.

### Fusion/fission classification

Protein clusters were classified as fusion, fission, or both based on the distribution of composite and syntenic split states, genome quality, taxonomic, and functional affiliation, using a simple heuristic approach. First, the frequency of split protein sets per family was used as an initial criterion for the class separation (less than 0.1 for fission, more than 0.9 for a fusion). The second criterion was the taxonomic distribution of composite proteins in bacteria. A fission assignment would prevail if the composite state was present in more than two lineages (phylum) or at least 1,000 bacterial genomes. On the other hand, the absence of bacterial diversity favored a fusion classification. The presence of composite and split proteins from closed genomes, the frequency in the case of split and composite proteins, was the third criterion to classify a protein cluster. When the majority of complete genomes in a protein family contained composite genes, the classification would favor fissions. On the opposite, the prevalence of split proteins in high-quality genomes would favor fusions. When available, an additional criterion was considering the median KO model coverage of split and composite proteins. In cases where fused proteins had full KO coverage and unfused proteins had it halved, this would indicate a fission. However, as many sequences used to build models are of bacterial origin, other criteria had the advantage. If the majority of criteria agreed either on fusion or on fission, then the assignment would be “high confidence,” otherwise a protein cluster would get the “probable” one. If no features were found to support either of the assignments a protein cluster would remain unclassified. Finally, if there is a high frequency of proteins that are composite and split partners mapped to more complex proteins within the same cluster then the assignment would be “fusion and fission.” Furthermore, all protein families were manually analyzed.

## Data Availability

The mappings are at the basis of Fig. 1 to 5; Fig. S1 to 3 are available in supplemental tables. Bacterial mappings are deposited at Figshare (54) ( https://doi.org/10.6084/m9.figshare.24084189). The code will be made available upon request.

## SUPPLEMENTAL MATERIAL

Supplemental Information msystems.00948-23-s0001.pdf Supplemental discussion, supplemental figures, and captions for supplemental tables.

Table S1 msystems.00948-23-s0002.xlsx Archaeal data set: taxonomy, completeness, and contamination.

Table S2 msystems.00948-23-s0003.xlsx Archaeal fission/fusion families distribution and functional annotations of the composite and split components, including domain assignments.

Table S3 msystems.00948-23-s0004.xlsx Bacterial data set: taxonomy, completeness, and contamination.

Table S4 msystems.00948-23-s0005.xlsx *E. coli* fusions in archaea.

Table S5 msystems.00948-23-s0006.xlsx Presence or absence of fusion/fission composite proteins per assembly.

Table S6 msystems.00948-23-s0007.xlsx Counts of fusion/fission families per taxonomic lineage.

Table S7 msystems.00948-23-s0008.xlsx Presence or absence of archaeal composite proteins and corresponding bacterial mappings.

ASM does not own the copyrights to Supplemental Material that may be linked to, or accessed through, an article. The authors have granted ASM a non-exclusive, world-wide license to publish the Supplemental Material files. Please contact the corresponding author directly for reuse.
