# Supplementary material for: Fusion/fission protein family identification in Archaea
Source: mSystems. 2024 May 3;9(6):e00948-23. doi: 10.1128/msystems.00948-23 (PMC11237513; doi:10.1128/msystems.00948-23)
Supplement: Supplemental Information — Supplemental discussion, supplemental figures, and captions for supplemental tables. [file msystems.00948-23-s0001.pdf]

Supplementary information

Fusion/Fission Protein Families Identification in Archaea

[a] Genome Evolution and Ecology Group, Department of Functional and Evolutionary Ecology, University of Vienna, Austria

*present address: NutriMo Pets, LLC, Houston, Texas, USA, govind.nair@nutrimopets.com.

Running Head: archaeal fussion/fission protein families

Address correspondence to Filipa L. Sousa, filipa.sousa@univie.ac.at

This supplementary material contains supplementary discussion, supplementary figures and legends of supplementary tables.

Supplementary Discussion

Genetic information processing

Within the genetic information processing category, the fusion/fission protein families functional annotations cover a range of DNA, RNA and protein processing pathways, with more than 30 protein clusters involved in DNA repair and replication alone. Although in these, fission events tend to prevails, several families, demonstrate more complicated patterns. This is the case of DNA polymerase B and DNA helicase Mcm protein families (classified as fissions) containing inteins. Inteins are part of the auto-spliced gene as the protein is expressed[1]. Insertion of inteins can happen multiple times and eventually, lead to fission events between or within domains, which produce misleading functional annotations. In addition, two fusion events containing the uracil-DNA glycosylase domain were identified in unclassified *Euryarchaeota* and *Thermoplasmatales*. Furthermore, new insights on eucaryotic like DNA repair endonuclease XPF protein evolution can be provided through the identification of the corresponding split protein pairs within the DPANN superphyla mapping to *Euryarchaeota* composite proteins, that suggest the loss of the protein in *Crenarchaeota*.

Within transcription processes, less than ten fusion/fission events were identified. The two largest protein clusters from this category correspond to the transcription initiation factor TFB and the RNA polymerase subunit B. The first has sixteen split pairs and according to its taxonomic distribution and the domain architecture of the distantly homologous eukaryotic form[2], might represent a fission event. In the case of the cluster containing RNA polymerase B proteins, on the contrary, several composite proteins corresponding to a fusion of the two subunits (rpoB1 and rpoB2) are found, and this cluster assigned as fusion. This composite form is mostly present in *TACK* and *Asgard* supergroups, while the split forms are widely distributed in *Euryarchaeaota*. The identified clusters associated with translation correspond to ribosomal proteins, ribosome biogenesis proteins and aminoacyl-tRNA biogenesis proteins. Clusters from the first two categories are predominated classified as fission. Nevertheless, we observed two singleton fusion events with high support from the conservation of the respective syntenic split pairs. The First corresponds to a fusion between the large ribosomal subunit protein L5 and the small ribosomal protein S4e, identified in many TACK and Euryarchaeota lineages as well as *Ca*. Aenigmarchaeota (*DPANN*). The second is a fusion between the small ribosomal protein S3 and the ribonuclease P protein subunit POP4, observed in single *Ca.* Bathyarchaeota assembly. As for tRNA biogenesis, clusters containing synthetases or with methyltransferases involved in amino acid metabolism represent taxonomic or assembly restricted fission/fusion

events, are only identified in taxonomically unclassified lineages. The fusion partners of these enzymes are either cytochrome-containing or sulfur carrier proteins, reflecting perhaps, the recruitment of the module to perform other functions associated with group-transfers.

In the archaeosine synthase alpha-subunit protein family, we observe that the composite protein is present in *Euryarchaeaota* and *Asgards*, while in *Crenarchaeota* representatives the split versions of the protein is present. Within the protein folding and degradation category, a few clusters corresponding to fission events were found. However, we identified a singleton fusion event between a proteosome subunit and an uncharacterised universal archaeal protein in a *Nitrosopumilus* assembly. This fusion event has high support from the split protein side and hints for the possible involvement of the composite protein in folding processes.

Classification of complex protein families

The classification of a fusion/fission event can be complicated by the existence of homologous protein in the same cluster. For example, in the probable fusion cluster containing ABC transporters (cluster 19), several homologous are present. In this case, the fusion of two ATP-binding domains corresponds to the three functionally distinct proteins: the energy-coupling factor transporter (EcfA1 and EcfA2)[3], the general nucleoside transporter[4] and the methyl-coenzyme M reductase system (component A2) [5]. A final classification problem is the recurring lack of agreement between bacterial and archaeal domains on the fission/fusion state of a protein family. One of such cases refers to a fusion/fission event of hydroxymethylpyrimidine kinase/phosphomethylpyrimidine kinase, ThiD, and thiamine-phosphate diphosphorylase (ThiN), involved in thiamine metabolism, a vitamin ubiquitous in prokaryotes[6]. In Bacteria, phosphorylation of the pyrimidine and condensation with thiazole is catalyzed by two separate enzymes, ThiD and ThiE (analogous to ThiN)[7,8], with a small fraction of organisms containing a sequence resulting from a fusion of the two. In *Archaea*, on the other hand, the majority of organisms has the composite version of the enzymes (ThiDN), with only 19 syntenic pairs of split proteins found across different phyla, rather suggesting one or more fission events.

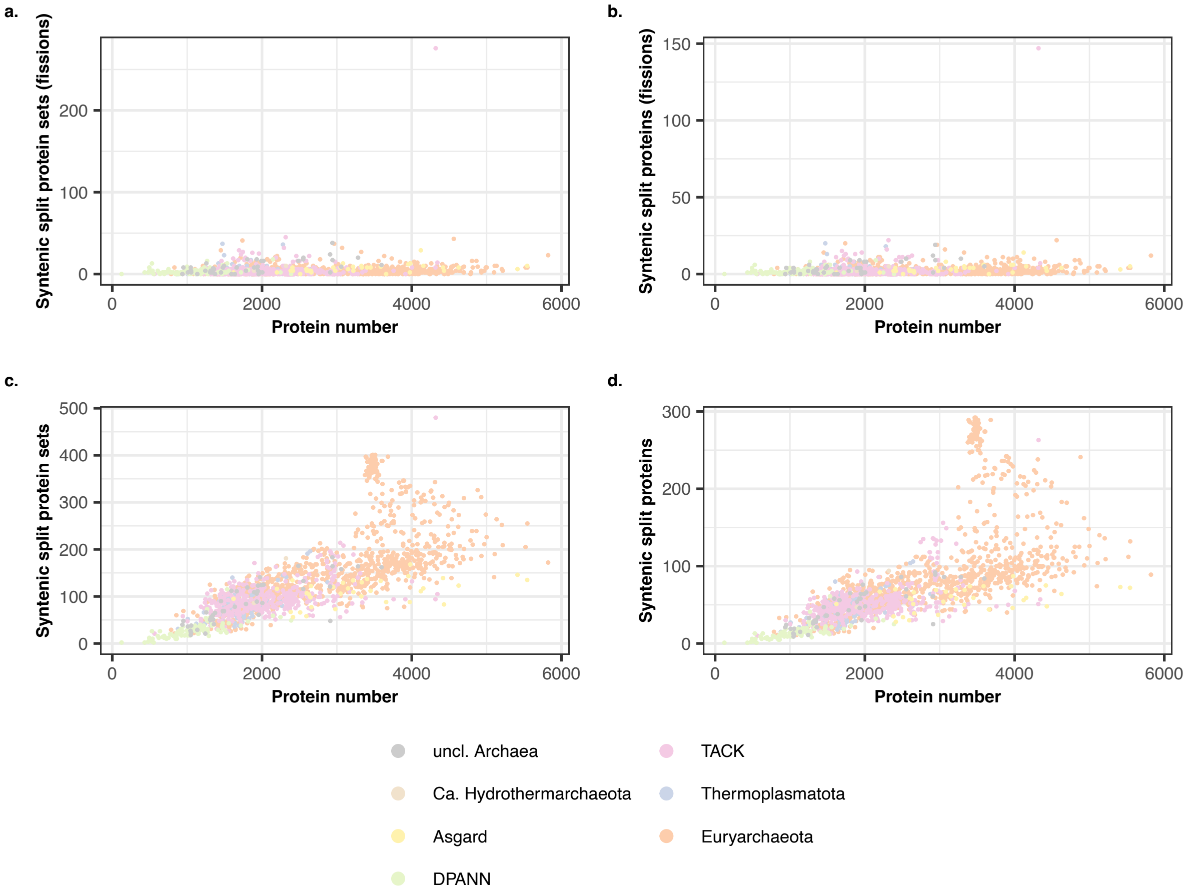

Fig S1 – Relation between split proteins (or split sets) and total number of proteins per assembly. a) Correlation between the number of high-confidence fission split sets and number of proteins per assembly. b) Correlation between the number of high-confidence fission split proteins and number of proteins per assembly. c) Correlation between total number of split sets and number of proteins per assembly. d) Correlation between the total number of split proteins and number of proteins per assembly.

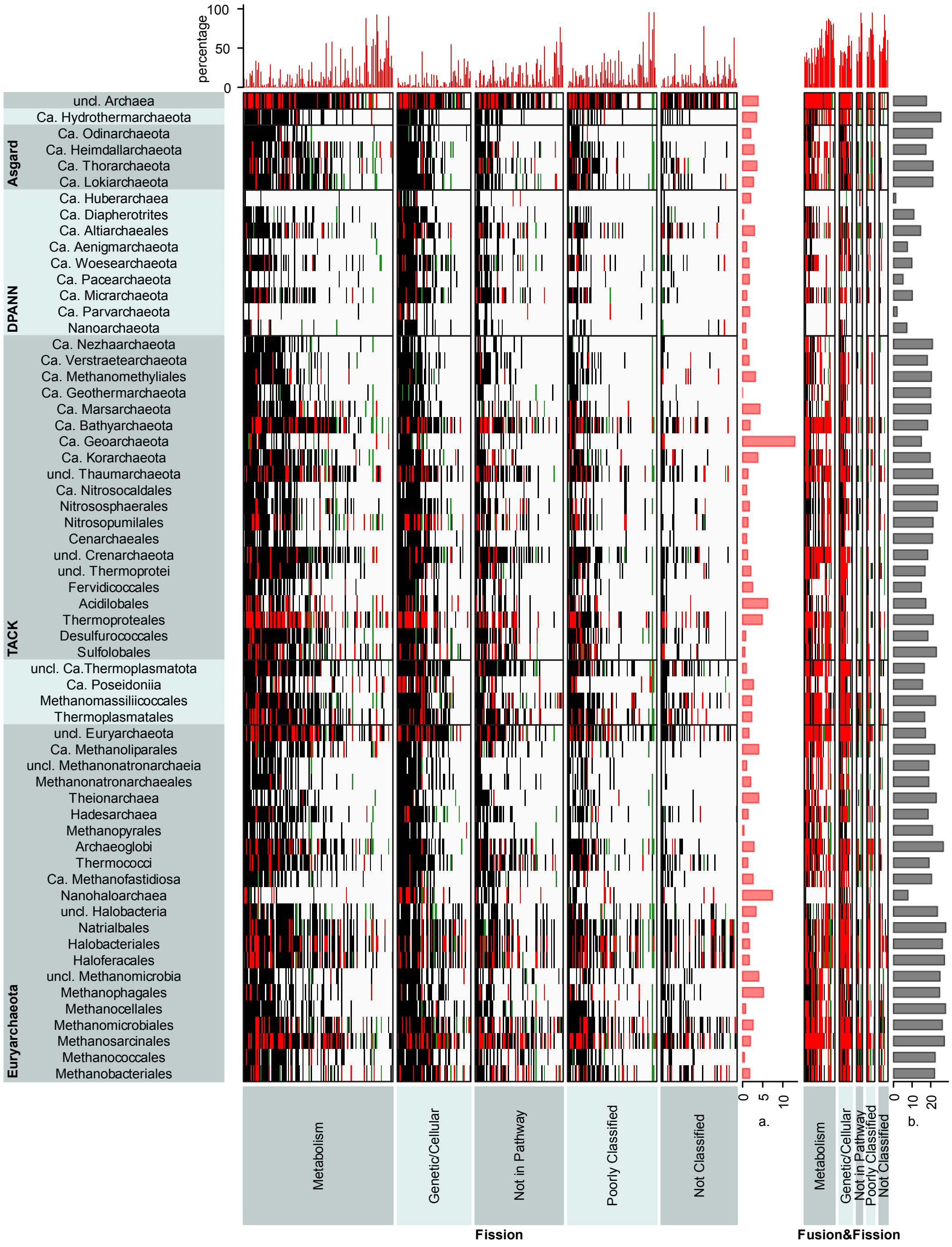

Fig S2 Taxonomic distribution of fissions across 1678 archaeal assemblies. The taxonomic level, represented on the vertical axis, is grouped by order, phyla or superphyla (indicated in bold). Protein clusters/families are represented on the horizontal axis, with functional category indication underneath. High confidence fissions are represented on the left side, with fission-fusion families on the right side. Red indicates presence of split syntenic sets, green of split sets (where syntenic representatives are absent), black of composite proteins, and white absence of any within the taxonomic rank. Top bar chart shows the percentage of split proteins (from syntenic sets) over total number of proteins per family is shown. Singletons were excluded from the figure. On the right, the row annotation bar charts show a. the average

number of fission events per genome (split protein count); b. the average number of “fusion and fission” events per genome (composite protein count);

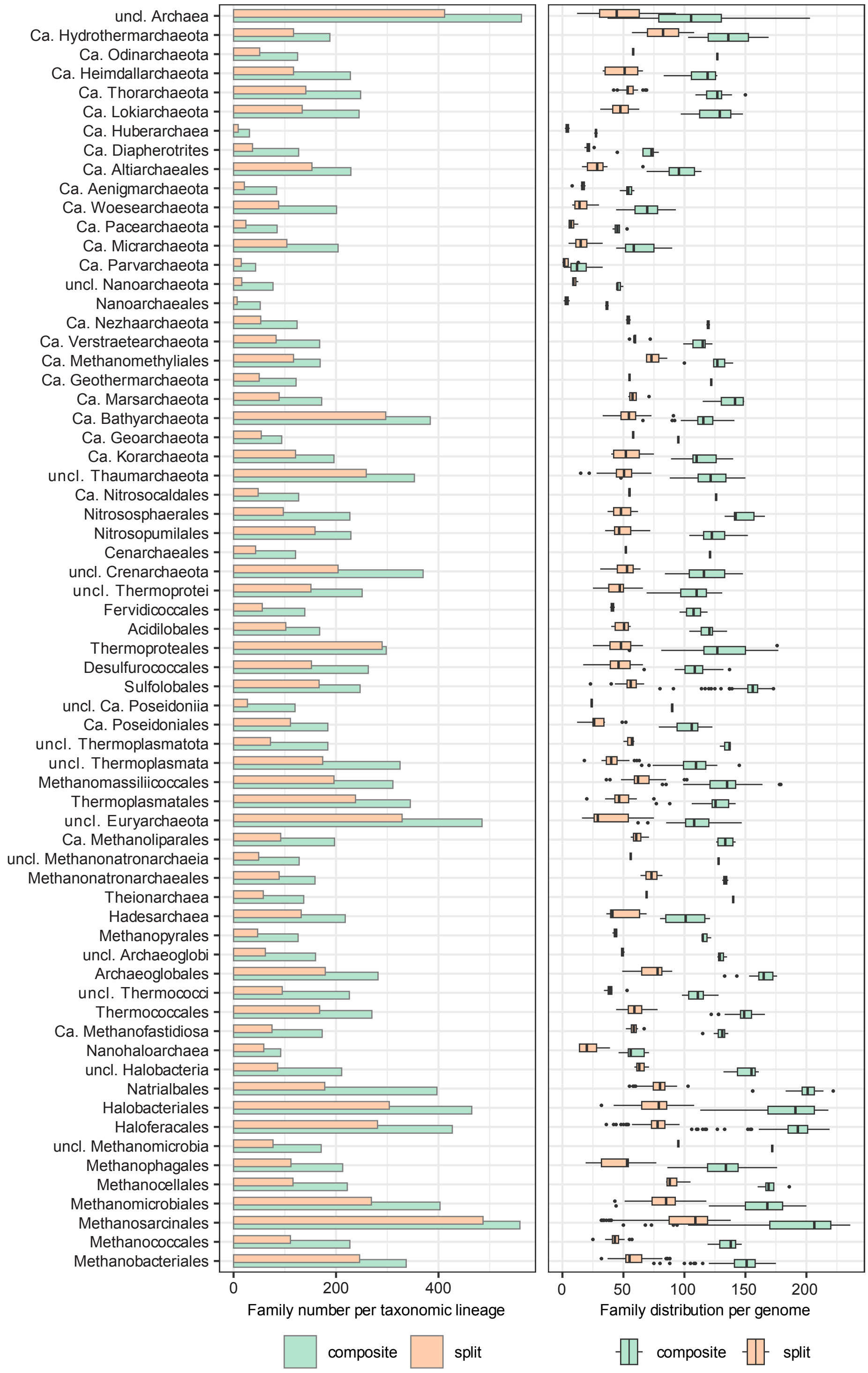

Fig S3. Presence and distribution of composite and split proteins over archaeal taxonomic lineages (order or higher). The bar chart to the left indicates the total counts of the fusion/fission families in split state (for syntenic sets only) and for the composite side. The box plot to the right shows the distribution of the fusion/fission families within the lineage representatives (assemblies).

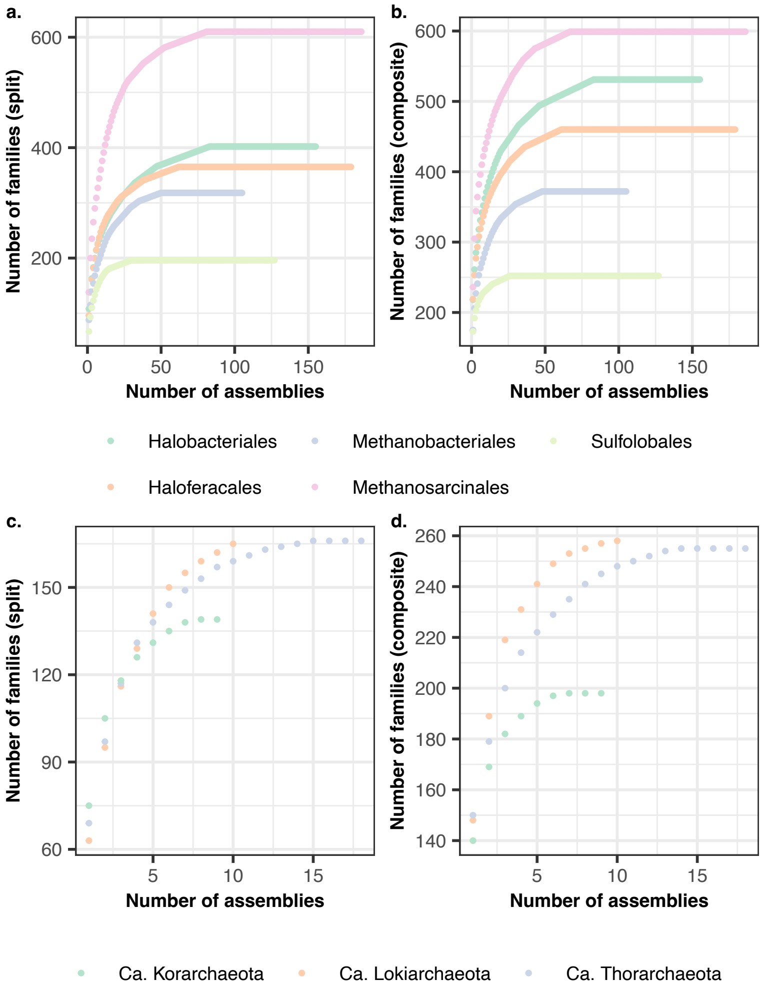

Fig S4. Gain in number of fusion/fission families with the addition of each assembly per lineage (order or higher). **a.** Gain in number of fusion/fission families for the syntenic splits in well-represented lineages. **b.** Gain in number of fusion/fission families for the composite state in well-represented lineages. **c.** Gain in number of fusion/fission families for the syntenic splits in poorly represented lineages. **d.** Gain in number of fusion/fission families for the composite state in poorly represented lineages.

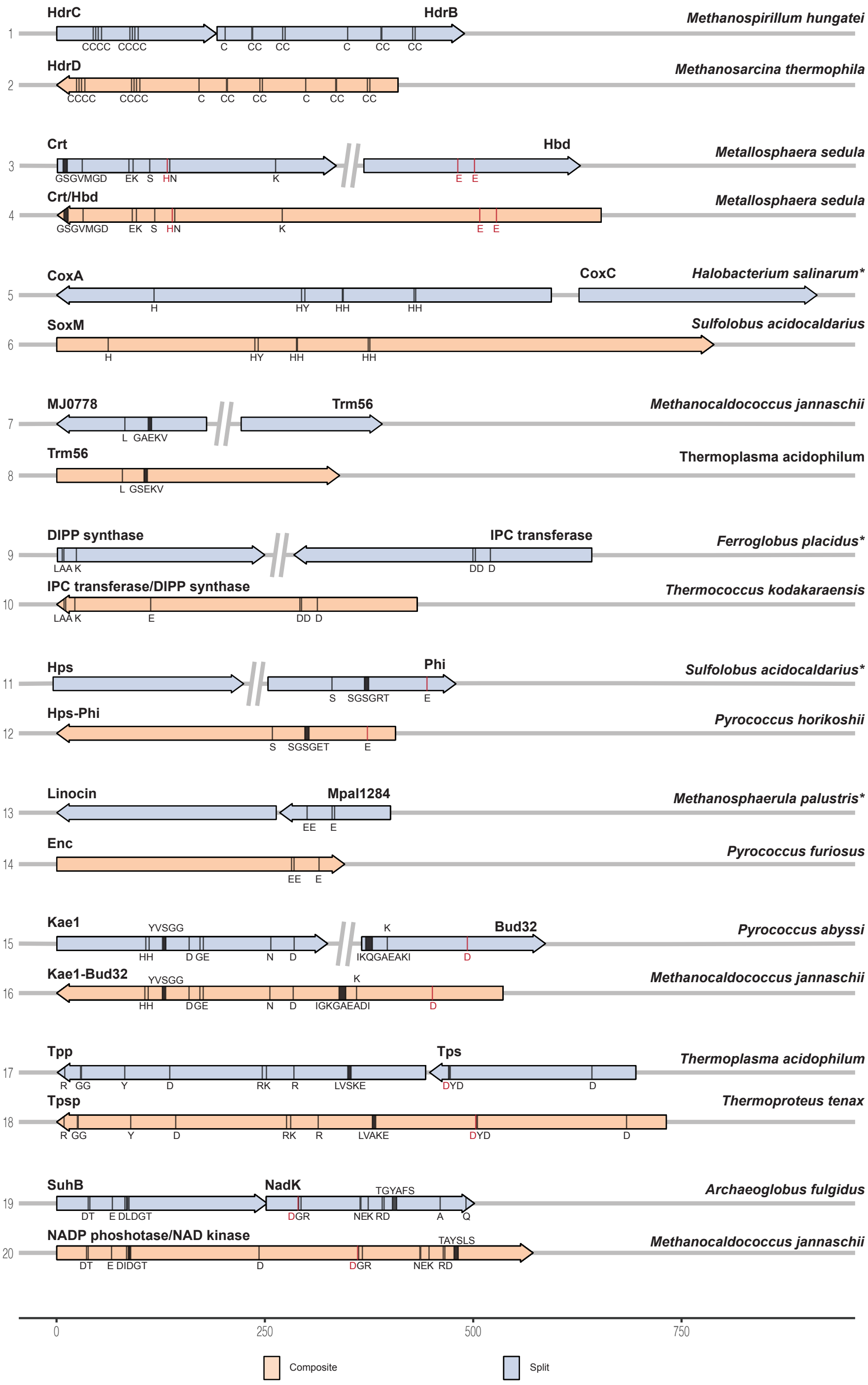

Fig S5. Experimentally validated representatives of fusion/fission families in the composite (orange) and split (blue) states. Binding cites/residues marked in black; active cites/residues marked in red. Star next to species name indicates that not all proteins were experimentally validated. The direction preserved from genomic data, if possible, breaks in lines show that encoding genes are not in synteny, scale on the bottom indicates length in amino acids. **Hdr**: CoB--CoM heterodisulfide reductase**, HdrB:** subunit B**, HdrC:** subunit C**, HdrD:** subunit D**; Crt:** crotonase**; Hbd:** 3-hydroxybutyryl-CoA dehydrogenase**; CoxA:** cytochrome c oxidase subunit 1**; CoxC:** cytochrome c oxidase subunit 3**; SoxM:** quinol oxidase subunit 1/3**; MJ0778:** uncharactarised protein with phosphohydrolase domain; **Trm56**: tRNA

(cytidine(56)-2'-O)-methyltransferase**;** **DIPP:** di-myo-inositol-1,3'-phosphate-1'-phosphate; **IPC**: 1L-myo-inositol 1-phosphate cytidylyl; **HxlB**: 3-hexulose 6-phosphate synthase; **Phi**: 3-hexulose-6-phosphate isomerase; **Mpal1284**: uncharacterised protein with rubrerythrin domain; **Enc**: encapsulin nanocompartment shell protein; **Kae1**: tRNA N6-adenosine threonylcarbamoyltransferase**;** **Bud32:** TP53 regulating kinase; **Tpp**: trehalose-6-phosphate phosphatase; **Tps**: trehalose-6-phosphate synthase; **Tpsp**: trehalose-6 phosphate synthase/phosphatase; **SuhB**: fructose-1,6-bisphosphatase/inositol-1-monophosphatase**;** **NadK:** NAD kinase**.**

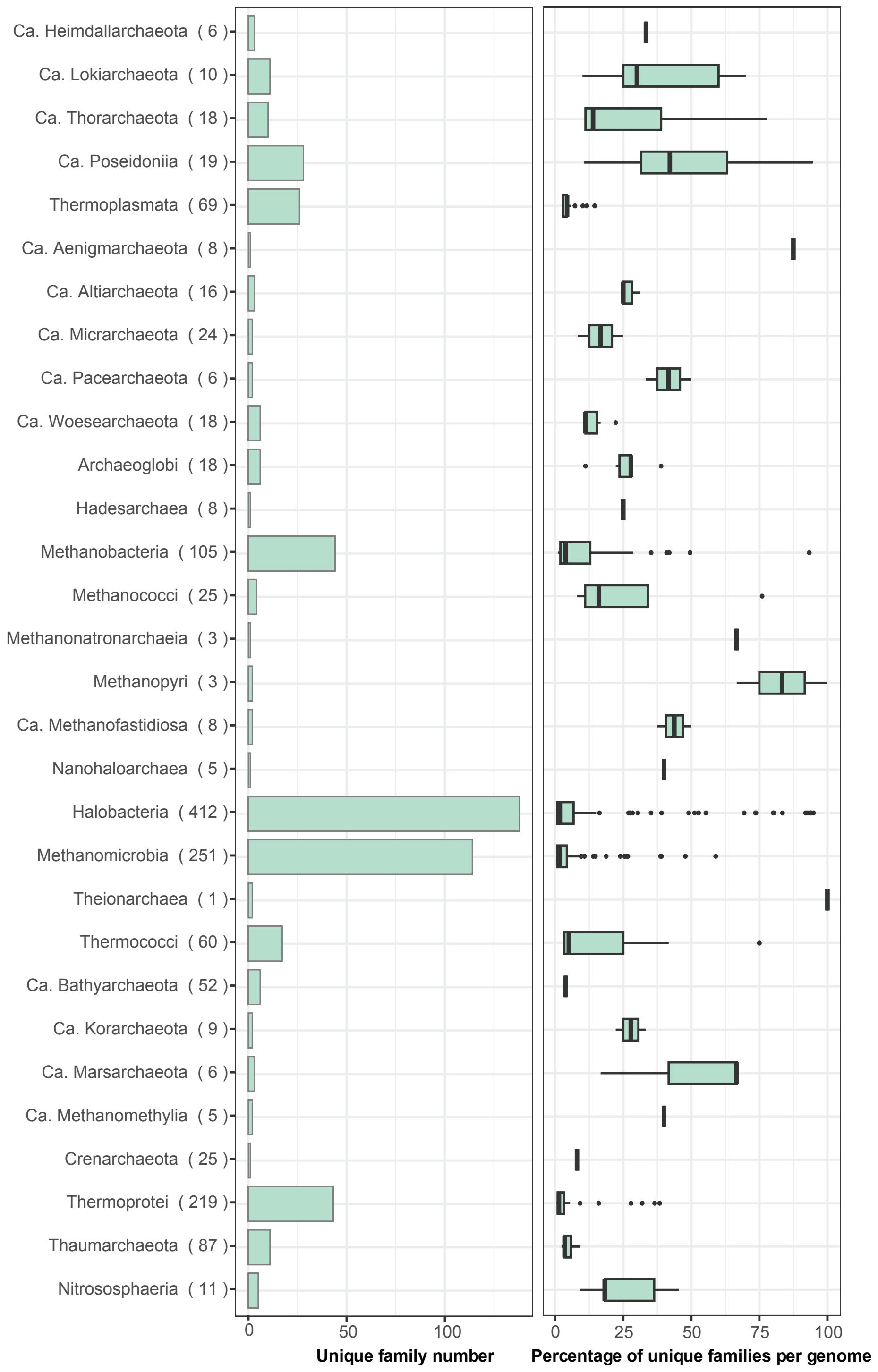

Fig S6. Presence and distribution of lineage-specific fusion/fission families in the composite state (class or higher). The bar chart to the left indicates the total counts of the fusion/fission families for the composite proteins. The box plot to the right shows the distribution of the fusion/fission families within the lineage representatives (assemblies).

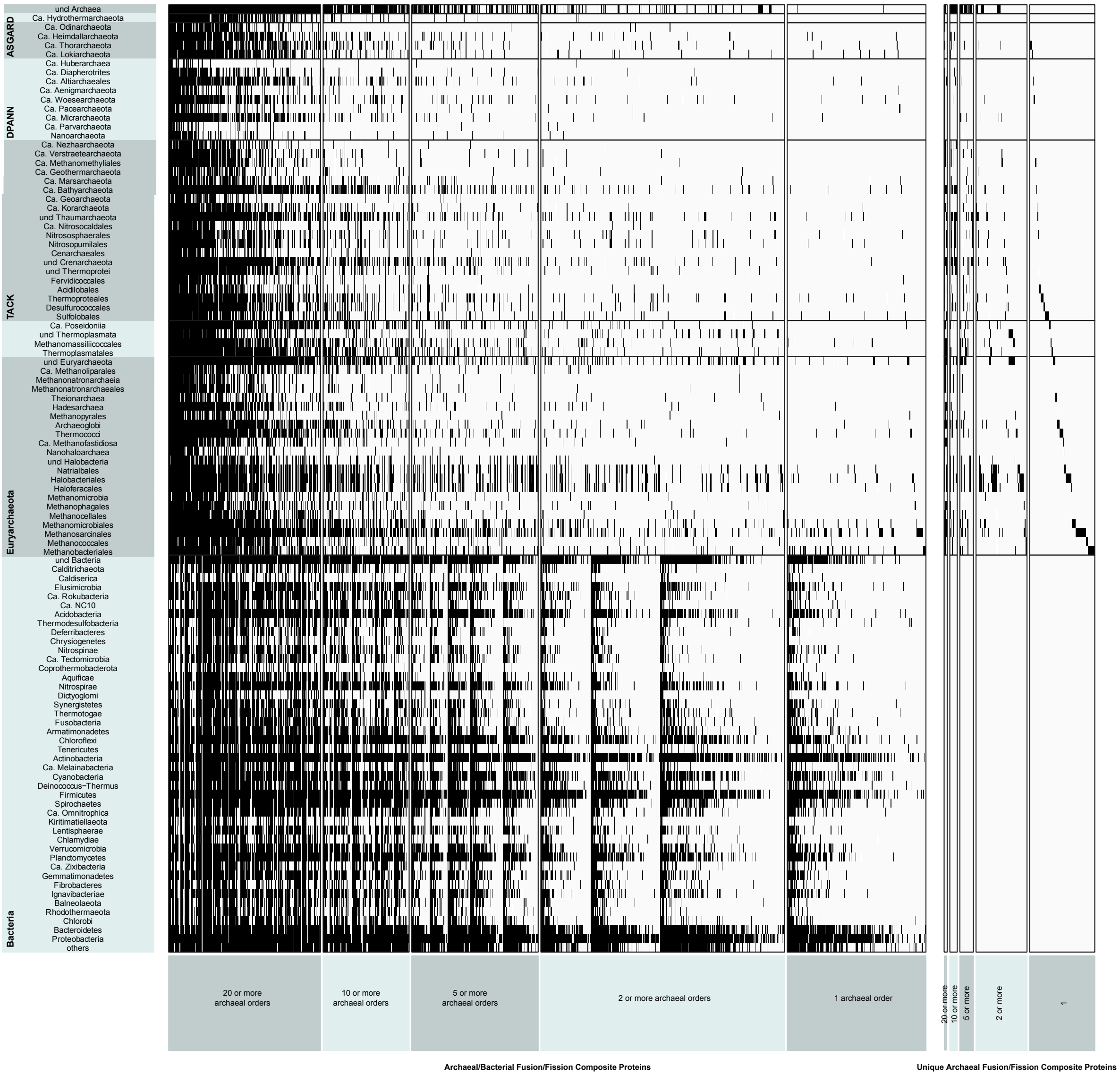

Fig S7. Bacterial mappings of composite proteins to archaeal fusion/fission families. The taxonomic level, represented on the vertical axis, is grouped by order, phyla or superphyla (indicated in bold). Protein clusters/families are represented on the horizontal axis, with number of archaeal groups in which the family is present indicated at the bottom. Black indicates presence, and white absence of the protein within the taxonomic rank. Singletons were excluded from the figure.

Supplementary Table Legends

Table S1. Archaeal dataset taxonomy, completeness and contamination.

Table S2. fussion fission (sheet 1): Archaeal fission/fusion families distribution and functional annotations of the composite and split components, including domain assignments.

singleton (sheet 2): Archaeal fission/fusion families distribution and functional annotations of the composite and split components in singletons, including domain assignments

Table S3. Bacterial dataset taxonomy, completeness and contamination.

Table S4. **E. coli fusions in archaea** (sheet 1): Curated set of fusions in E. coli (Henry C, et all 2016) and its presence in Archaea. Protein families present in the dataset, but not identified in screening have either limited distribution of split or composite states and filtered by coverage/identity thresholds. Due to lack of syntenic split components, protein families can be classified as fissions. Apart from domain anotations, KEGG and TCDB(for transporters) was used in assignments. **archaea fusions** (sheet 2)**:** Experimentally validated archaeal fused proteins.

Table S5. Presence/absence of fusion/fission composite proteins per assembly.

Table S6. Counts of fusion/fission families per taxonomic lineage. That includes total counts, average counts of fusion/fission families per genome, average counts of genomes per fusion/fission family.

Table S7. Presence/absence of archaeal composite proteins and corresponding bacterial mappings.

Bibliography

1. Gogarten, J. P., Senejani, A. G., Zhaxybayeva, O., Olendzenski, L. & Hilario, E. Inteins: Structure, function, and evolution. *Annu. Rev. Microbiol.* **56**, 263–287 (2002).
2. Thomm, M. Archaeal transcription factors and their role in transcription initiation. *FEMS Microbiol. Rev.* **18**, 159–171 (1996).
3. Karpowich, N. K. & Wang, D. N. Assembly and mechanism of a group II ECF transporter. *Proc. Natl. Acad. Sci. U. S. A.* **110**, 2534–2539 (2013).
4. Martinussen, J., Sørensen, C., Jendresen, C. B. & Kilstrup, M. Two nucleoside transporters in Lactococcus lactis with different substrate specificities. *Microbiology* **156**, 3148–3157 (2010).
5. Kuhner, C. H., Lindenbach, B. D. & Wolfe, R. S. Component A2 of methylcoenzyme M reductase system from Methanobacterium thermoautotrophicum ΔH: Nucleotide

sequence and functional expression by Escherichia coli. *J. Bacteriol.* **175**, 3195–3203 (1993).

6. Morett, E. *et al.* Systematic discovery of analogous enzymes in thiamin biosynthesis. *Nat. Biotechnol.* **21**, 790–795 (2003).
7. Vander Horn, P. B., Backstrom, A. D., Stewart, V. & Begley, T. P. Structural genes for thiamine biosynthetic enzymes (thiCEFGH) in Escherichia coli K-12. *J. Bacteriol.* **175**, 982–992 (1993).
8. Mizote, T., Tsuda, M., Smith, D. D. S., Nakayama, H. & Nakazawa, T. Cloning and characterization of the thiD/J gene of Escherichia coli encoding a thiamin-synthesizing bifunctional enzyme, hydroxymethylpyrimidine kinase/phosphomethylpyrimidine kinase. *Microbiology* **145**, 495–501 (1999).
